# Mitotic Centromere-Associated Kinesin (MCAK/KIF2C) Regulates Cell Migration and Invasion by Modulating Microtubule Dynamics and Focal Adhesion Turnover

**DOI:** 10.3390/cancers13225673

**Published:** 2021-11-12

**Authors:** Ha Hyung Moon, Nina-Naomi Kreis, Alexandra Friemel, Susanne Roth, Dorothea Schulte, Christine Solbach, Frank Louwen, Juping Yuan, Andreas Ritter

**Affiliations:** 1 Obstetrics and Prenatal Medicine, Department of Gynecology and Obstetrics, University Hospital Frankfurt, J. W. Goethe-University Frankfurt, Theodor-Stern-Kai 7, D-60590 Frankfurt, Germany; moonhahyung@gmail.com (H.H.M.); nina-naomi.kreis@kgu.de (N.-N.K.); alexandra.friemel@kgu.de (A.F.); Susanne.Roth@kgu.de (S.R.); Christine.Solbach@kgu.de (C.S.); Frank.Louwen@kgu.de (F.L.); yuan@em.uni-frankfurt.de (J.Y.); 2 Institute of Neurology (Edinger Institute), University Hospital Frankfurt, J. W. Goethe University, D-60528 Frankfurt, Germany; Dorothea.Schulte@kgu.de

**Keywords:** MCAK, microtubule dynamics, focal adhesion, migration, motility, invasion, plus-tip, actin cytoskeleton, depolymerization, CRISPR/dCas9

## Abstract

**Simple Summary:**

The microtubule (MT) cytoskeleton is a crucial factor for organized cell motility and migration of cancer as well as benign cells. Mitotic centromere-associated kinesin (MCAK/KIF2C) is a member of the kinesin-13 family, which is important for the regulation of MT dynamics. Its overexpression has been reported to be related to increased metastasis in various tumor entities. Our study further elucidate how MCAK’s is able to modulate cell migration and invasion. Interfering with the precise regulated expression of MCAK led to impaired FA protein composition and altered their phosphorylation status, disturbed the assembly and disassembly rate of FA, delayed cell adhesion, and compromised the plus-tip dynamics of MTs. MCAK regulates these processes by affecting the actin-MT cytoskeleton dynamics, providing molecular mechanisms by which a deregulation of MCAK could promote tumor metastasis.

**Abstract:**

The microtubule (MT) cytoskeleton is crucial for cell motility and migration by regulating multiple cellular activities such as transport and endocytosis of key components of focal adhesions (FA). The kinesin-13 family is important in the regulation of MT dynamics and the best characterized member of this family is the mitotic centromere-associated kinesin (MCAK/KIF2C). Interestingly, its overexpression has been reported to be related to increased metastasis in various tumor entities. Moreover, MCAK is involved in the migration and invasion behavior of various cell types. However, the precise molecular mechanisms were not completely clarified. To address these issues, we generated CRISPR/dCas9 HeLa and retinal pigment epithelium (RPE) cell lines overexpressing or downregulating MCAK. Both up- or downregulation of MCAK led to reduced cell motility and poor migration in malignant as well as benign cells. Specifically, it’s up- or downregulation impaired FA protein composition and phosphorylation status, interfered with a proper spindle and chromosome segregation, disturbed the assembly and disassembly rate of FA, delayed cell adhesion, and compromised the plus-tip dynamics of MTs. In conclusion, our data suggest MCAK act as an important regulator for cell motility and migration by affecting the actin-MT cytoskeleton dynamics and the FA turnover, providing molecular mechanisms by which deregulated MCAK could promote malignant progression and metastasis of tumor cells.

## 1. Introduction

Migration is not only essential for a variety of physiological processes, including the development of organisms, repair of damaged tissue and immune response, but also for multiple pathological events like tumor metastasis [1,2]. Migration requires the cytoskeleton, a complex and dynamic network consisting of three main components, actin microfilaments, intermediate filaments and microtubules (MTs) [3,4,5]. While the protein actin, the main driving factor for directed migration, initiates cellular protrusions important for the general force generation and the interaction with myosin to induce a contractile force at the leading edge of cells [6,7], the MT cytoskeleton is more passive as an essential part for the transport of new membrane components, mitochondria, chromosomes, secretory vesicles, signaling molecules and integrins, or as a crucial player for promoting integrin endocytosis and focal adhesion (FA) protein recycling [5,8,9].

Importantly, the actin- and the MT network operate together in an orchestrated manner [4,5,6]. MT growth triggers a positive feedback mechanism by stimulating T-lymphoma invasion and metastasis-inducing protein 2 (TIAM2) [10], which activates Rac family small GTPase 1 (RAC1) in FAs, inducing actin repolymerization and the formation of membrane protrusions [8]. The depolymerization of the MT cytoskeleton leads to stabilization of FAs and their anchoring complexes to the actin cytoskeleton [4,5,8]. These adhesion complexes, composed of around 2400 different proteins, are necessary for a proper signal transduction and force generation [11]. The main proteins linking the integrin transmembrane receptors to the actin cytoskeleton are mediated by the interactions of focal adhesion kinase (FAK)/paxillin, talin/vinculin and α-actinin/zyxin/VASP (vasodilator stimulated phosphoprotein) to ensure a proper directional migration [12]. The formation of stable FAs is initiated by a signaling cascade triggered by the binding of extracellular matrix (ECM) components to integrins, leading to the activation of FAK. This enables FAK to phosphorylate paxillin at tyrosine 118 and tyrosine 31, promoting docking sites for several FA proteins including vinculin, α-actinin, and talin, leading to the stabilization of newly formed FAs [13,14].

The mitotic centromere-associated kinesin (MCAK), the best studied member of the kinesin-13 family and encoded by the gene *KIF2C*, is important for the regulation of MT dynamics, prevalent in mitosis [15,16,17]. MCAK functions as highly potent MT depolymerase in a dimeric state. At the plus-end of MTs, MCAK triggers a conformational change of the MT protofilament, leading to the release of the terminal tubulin dimer bound to a MCAK dimer [16]. The deregulation of its activity leads to severe mitotic defects and chromosomal instability [15,16,18]. In line with the close relationship between the MT and actin cytoskeleton, we and others have shown that functional MCAK has an impact on cancer cell migration [19,20,21]. The inhibitory phosphorylation of Aurora B at serine 192 of MCAK affects the directed migration as well as the invasion capacity of colorectal and cervix carcinoma cell lines [19]. Moreover, it has been revealed that a signaling cascade of RAC1, Aurora B and MCAK facilitates polarization of endothelial cells [20], and that the activity of MCAK is crucial for positioning the centrosome towards the leading edge of cells, and the turnover of FAs [21]. To elucidate precisely how MCAK’s activity regulates the motility of cancer cells, using MCAK CRISPRi/a knockdown and overexpression cell lines, we show that interfering with the expression of MCAK causes a decreased cell motility and migration, which is attributed to an impaired FA turnover associated with compromised MT- and actin-cytoskeleton dynamics.

## 2. Materials and Methods

### 2.1. CRISPRi/A Cell Line Generation, Cell Culture and siRNA Transfection

The generation of HeLa/hTERT-RPE1 (RPE, hTERT-immortalized retinal pigment epithelial cell line) CRISPRi (MCAK knockdown, i for inhibition) and CRISPRa (a for activation) cell lines was performed as previously described [22] (the HeLa, MDA-MB-231 and hTERT RPE-1 cell lines were obtained from ATCC (Manassas, VA, USA)). In short, RPE/HeLa cell lines were stably transduced with a lentiviral vector expressing either dCas9-BFP-KRAB (CRISPRi) or with dCas9-SunTag_10x_v4_–P2A-BFP-NLS and scFv-GCN4-GFP-VP64 (CRISPRa). CRISPRa cells were then selected by transfection with a sgRNA targeting the transmembrane protein CXCR4, and highly expressing CXCR4 cells were isolated by FACS sorting and grown as a single cell clown for further analyses. After the establishment of RPE/HeLa CRISPRi/a cell lines by M. Jost et al. [22], the sgRNA protospacers targeting MCAK or a non-targeting control protospacer (sgcon) were cloned into the pCRISPRia-v2 vector (Addgene: #84832): KIF2C GGGCGGCGTTAAGACTTCGTA CRISPRi, KIF2C GCGTCTCCCCCAAGGCTCCGC CRISPRa, neg_ctrl-1 GAACGACTAGTTAGGCGTGTA CRIPSRi/a. The individual pCRISPRia-v2 sgMCAK(i/a) or sgcon vectors were packaged into lentivirus and transduced to stable HeLa/RPE CRISPRi/a cell lines. These cell lines were further selected using 10 mg/mL puromycin for 7 days, checked for their gene expression of MCAK (sgMCAK vs. sgcon), and subjected for functional assays.

MDA-MB-231, HeLa, and RPE cells were cultured as instructed by the supplier (ATCC, Wesel, Germany). A well-established siRNA targeting the 3′-untranslated region of human MCAK [19,23,24] was synthesized by Sigma-Aldrich (Taufkirchen, Germany), with the following sequence: 3′- CCG TTC CGT GAG AGC AAG CT -′5. Control siRNA was obtained from Qiagen (Hilden, Germany) and siRNAs (30 nM) were transiently transfected with Oligofectamine^TM^ (Thermo Fisher Scientific, Dreieich, Germany) as reported [25].

### 2.2. Western Blot and Immunofluorescence (IF) Staining

Cellular lysates were prepared using RIPA buffer (50 mM Tris pH 8.0, 150 mM NaCl, 1% NP-40, 0.5% Na-desoxycholate, 0.1% sodium dodecyl sulfate (SDS), 1 mM NaF, phosphatase, and protease inhibitor cocktail tablets (Roche, Mannheim, Germany)). Western blot analysis was performed as described [26] and the following antibodies were used: mouse monoclonal antibody against glyceraldehyde-3-phosphate dehydrogenase (GAPDH) (GTX627408) from GeneTex (BIOZOL, Eiching, Germany), mouse monoclonal antibody against cyclin B1 (#sc-245, Santa Cruz Biotechnology, Heidelberg, Germany), rabbit monoclonal antibody against Aurora A (#14475, Cell Signaling, Frankfurt, Germany), mouse monoclonal antibody against Polo-like kinase 1 (Plk1, #sc-17783, Santa Cruz Biotechnology, Heidelberg, Germany), mouse monoclonal antibody against anti-Kif2c (MCAK) (#sc-81305, Santa Cruz Biotechnology, Heidelberg, Germany) and mouse monoclonal antibody against β-actin (#a2228, Sigma-Aldrich, Taufkirchen, Germany).

For indirect immunofluorescence staining, cells were seeded on Nunc^TM^ Lab-Tek^TM^ SlideFlask chambers from Thermo Fisher Scientific (Schwerte, Germany). Cells were fixed for 8–10 min with methanol at −20 °C or with 4% paraformaldehyde containing 0.2% Triton X-100 for 15 min at room temperature. The following primary antibodies were used: rabbit polyclonal antibody against pericentrin (#ab28144, Abcam, Cambridge, UK), human immune serum against human anti-centromere (#HCT-0100, ImmunoVision, Springdale, Germany), mouse monoclonal FITC-conjugated antibody against α-tubulin (#F2168, Sigma-Aldrich, Taufkirchen, Germany), rabbit polyclonal antibody against α-tubulin (#ab15246, Abcam, Cambridge, UK), rabbit polyclonal antibody against FAK (#3285, Cell Signaling, Frankfurt, Germany), mouse monoclonal antibody against p-FAK (Y397) (#8556, Cell Signaling, Frankfurt, Germany), monoclonal mouse antibody against paxillin (#610052, BD Biosciences, Heidelberg, Germany) and rabbit monoclonal antibody against p-paxillin (Y118) (#2541, BD Biosciences, Heidelberg, Germany) and rat monoclonal antibody against active β1-integrin (CD29) (#9EG7, BD Pharmingen™, San Jose, CA, USA). FITC-, Cy3- and Cy5-conjugated secondary antibodies were obtained from Jackson Immunoresearch (Newmarket, UK). The actin cytoskeleton was stained by using phalloidin-TRITC (#P1951, Sigma-Aldrich, Taufkirchen, Germany) and DNA was visualized by using DAPI (4′,6-diamidino-2-phenylindole-dihydrochloride, Roche, Mannheim, Germany) staining.

Slides were examined using an AxioObserver.Z1 microscope (Zeiss, Göttingen, Germany) and images were taken via an AxioCam MRm camera (Zeiss, Göttingen, Germany). The stained slides were further examined by confocal laser scanning microscopy (CLSM) using Z-stack images with a HCXPI APO CS 63.0 x 1.4 oil objective (Leica CTR 6500, Heidelberg, Germany) in sequential excitation of fluorophores. A series of Z-stack images were captured at 0.5 μm intervals. All images in each experiment were taken with the same laser intensity and exposure time. Representatives shown in figures are generated by superimposing (overlay) individual images from confocal Z-sections. Fluorescence intensity was measured using ImageJ (National Institutes of Health, Bethesda, MD, USA). The fluorescence intensity of FA proteins were measured using ImageJ and calculated as described (1):

(1) $$FA\;protein\;intensity=\frac{\mathrm{protein}\;\mathrm{mean}\;\mathrm{gray}\;\mathrm{intensity}}{\mathrm{background}\;\mathrm{mean}\;\mathrm{gray}\;\mathrm{intensity}}$$

### 2.3. RNA Extraction and Real-Time PCR

Total RNAs of RPE, HeLa and MDA-MB-231 cells were extracted with EXTRACTME TOTAL RNA KIT (7 Bioscience GmbH, Freiburg, Germany). Reverse transcription was performed using GoScript™ Reverse Transcription Mix, Random Primers (Promega GmbH, Walldorf, Germany). Real-time PCR was performed with a StepOnePlus Real-time PCR System (Applied Biosystems, Darmstadt, Germany). The data were analyzed using StepOne Software v.2.3 (Applied Biosystems, Darmstadt, Germany). Using the comparative CT method, the gene expression was represented as ΔCT, which is normalized to GAPDH as endogenous control and is inversely related to the amount of target molecules in the reaction. The final result is presented as relative quantification (RQ) in mean with minimum and maximum range, indicating the difference in gene expression level between the analyzed samples, by setting the expression value of control condition as 1. As the RQ of a group is defined as 2−(ΔCTgroup−ΔCTcontrol), the RQ value for the control group itself leads to the value RQ = 1 without variation.

All probes for gene analysis were obtained from Applied Biosystems (Darmstadt, Germany): KIF2C (#Hs00901710_m1) and GAPDH (#Hs02758991_g1).

### 2.4. Cell Cycle, Cell Viability, Motility, Migration and Invasion

For cell cycle evaluation, cells were harvested, washed with PBS, fixed with chilled 70% ethanol for 30 min at 4 °C, treated with 1 mg/mL of RNase A (Sigma-Aldrich, Taufkirchen, Germany) and stained with 100 μg/mL of propidium iodide (PI) for 30 min at 37 °C. DNA content was determined by flow cytometric analyses (BD FACSCalibur™ Flow Cytometer).

Cell proliferation assays were performed using Cell Titer-Blue^®^ Cell Viability Assay (Promega, Mannheim, Germany) as described [27]. HeLa and RPE CRISPRi/a cells were seeded in 96-well plates and cultured for 0, 24, 48, 72 and 96 h (HeLa: 1500 cells; RPE: 2500 cells). Cell viability was measured at indicated time points.

For motility assays, cells were seeded into 24-well plates with a low confluence and were imaged for 12 h at 5 min time intervals. All time-lapse imaging was performed with an AxioObserver.Z1 microscope (Zeiss, Göttingen, Germany), imaged with an AxioCam MRc camera (Zeiss, Göttingen, Germany) equipped with an environmental chamber to maintain proper environmental conditions (37 °C, 5% CO_2_). The time-lapse movies were analyzed by using ImageJ 1.49i software (National Institutes of Health, Bethesda, MD, USA) with the manual tracking plugin, and Chemotaxis and Migration Tool (Ibidi GmbH, Germany). Tracks were derived from raw data points and were plotted in GraphPad Prism 7 (GraphPad software Inc., San Diego, CA, USA). The accumulated distance was calculated by using the raw data points by the Chemotaxis and Migration Tool. At least thirty random cells per experiment were analyzed and the experiments were repeated independently three times. The patterns of motility were evaluated as descripted previously [28]. The accumulated distance was approximated as the sum of all euclidian distances between the successive points. The migration velocity was calculated as the mean sum of all accumulated distances divided by the migration time. The unit is given in micrometers per minutes.

Cell migration/wound healing assays were performed with culture inserts from Ibidi (Ibidi GmbH, Martinsried, Germany). Culture inserts (cell-free gap of 500 μm) were placed in a 6-cm culture dish, and both wells were filled with cell suspensions (HeLa, 6.0 × 10^4^; MDA-MB-231, 7.5 × 10^4^). After 14 h, the culture inserts (in triplicate for each condition) were removed and the images were obtained at indicated time points. Cell-free area was evaluated based on bright-field images using the Axiovision SE64 Re. 4.9 software (Zeiss, Jena, Germany). For each experiment, at least four migration front images were taken and analyzed, and the experiments were independently performed three times.

For invasion assay, cells were seeded in 24-well transwell Matrigel chambers according to the manufacturer’s instructions (Corning GmbH, Kaiserslautern, Germany). Briefly, cells (MDA-MB-231, 7.0 × 10^4^; HeLa, 6.0 × 10^4^) were seeded into the upper chamber of the transwell in 500 μL serum-free medium and the lower chamber was filled with 750 μL serum-free medium. After 12 h, the medium of the lower chamber was discarded and the invasion assays were started by adding medium containing 10% FBS for the next 24 h. Cells were fixed with ethanol and stained with DAPI. Invaded cells were counted with a microscope. The experiments were independently performed three times.

### 2.5. Cell Spreading/Adhesion, Actin Repolymerization and Nocodazole Washout Assay

For cell spreading/adhesion assays, four-chamber slides were coated with 2 µg/mL fibronectin for 60 min (Merck Millipore, Darmstadt, Germany). Cells were trypsinized, reseeded on fibronectin-coated slides (HeLa, 3.5 × 10^4^, MDA-MB-231 and RPE, 4.5 × 10^4^ cells per chamber), and incubated at 37 °C for 20 and 60 min. Non-adherent cells were removed by two careful washing steps with PBS and adherent cells were counted. Adherent cells were fixed with 4% paraformaldehyde containing 0.2% Triton X-100 and stained. Quantification of the cell area from slides released at 20 and 60 min post-plating were analyzed using ImageJ.

To evaluate the dynamics of the actin cytoskeleton, cells were starved in serum-free medium for 12 h. Cells were treated with latrunculin B (200 nM, BIOMOL GmbH, Hamburg, Germany) for 90 min to depolymerize actin fibers. The drug was washed out with PBS and the cells were released into serum-containing medium for 30 and 60 min. The cells were fixed with 4% paraformaldehyde containing 0.2% Triton X-100 for 15 min at room temperature before processing for immunofluorescence staining.

To analyze the dynamics of FAs, cells were treated with nocodazole (10 µM; Sigma-Aldrich, Taufkirchen, Germany) for 5 h to depolymerize MTs. The drug was washed out with PBS, and MTs were repolymerized in warm medium for indicated time points (0, 10, 30, 60 and 75 min). The cells were fixed and processed for immunofluorescence staining.

### 2.6. FA Turnover and EB3 (End Binding Protein 3) Tracking

For the analysis of single FAs in living cells, CRISPRi/a HeLa cells were transfected with RFP (red fluorescent protein)-paxillin for 24 h and seeded at 5 × 10^5^ cells/mL into FRAP (fluorescence recovery after photobleaching)-chambers. The cells were attached for at least 12 h to the chambers, and time lapse videos were taken for up to 6 h. The images were examined using an AxioObserver.Z1 microscope (Zeiss, Göttingen, Germany) and were taken using an AxioCam MRm camera (Zeiss, Göttingen, Germany) with Axiovision SE64 Re. 4.9 software (Zeiss, Jena, Germany). For each experiment, at least 5 images were taken and 30 FAs were analyzed with ImageJ (National Institutes of Health, Bethesda, MD, USA). The experiments were independently performed three times.

The comet tracking assay was performed with HeLa and RPE CRISPRa/i cells transfected with RFP-EB3, a MT plus-tip tracking protein, re-seeded in FRAP-chambers, and time-lapse microscopy was performed up to 5 min with a camera speed at maximum velocity. The videos were analyzed with the plugin plusTipTracker for Matlab (MathWorks^©^ (Natick, MA, USA)) as described [29]. For each experiment, at least 10 cells were imaged and around 1.500 to 4.000 EB3 comets were analyzed in each cell. The experiments were independently performed three times.

### 2.7. Statistical Analysis

Student’s *t*-test (two-tailed and paired or homoscedastic) was used to evaluate the significance of difference between diverse groups for gene analysis, cell viability assay, cell cycle distribution and phenotype analyses. The statistical evaluation of the single cell tracking assay, FA protein analyses, time-lapse microscopy analyses and cell adhesion analyses were performed by using an unpaired Mann–Whitney *U* test (two-tailed). Difference is considered as statistically significant when *p* < 0.05.

## 3. Results

### 3.1. Characterization of HeLa/RPE CRISPR/dCas9 sgMCAK Knockdown (CRISPRi) and Overexpression (CRISPRa) Cell Lines

**Figure 1:**
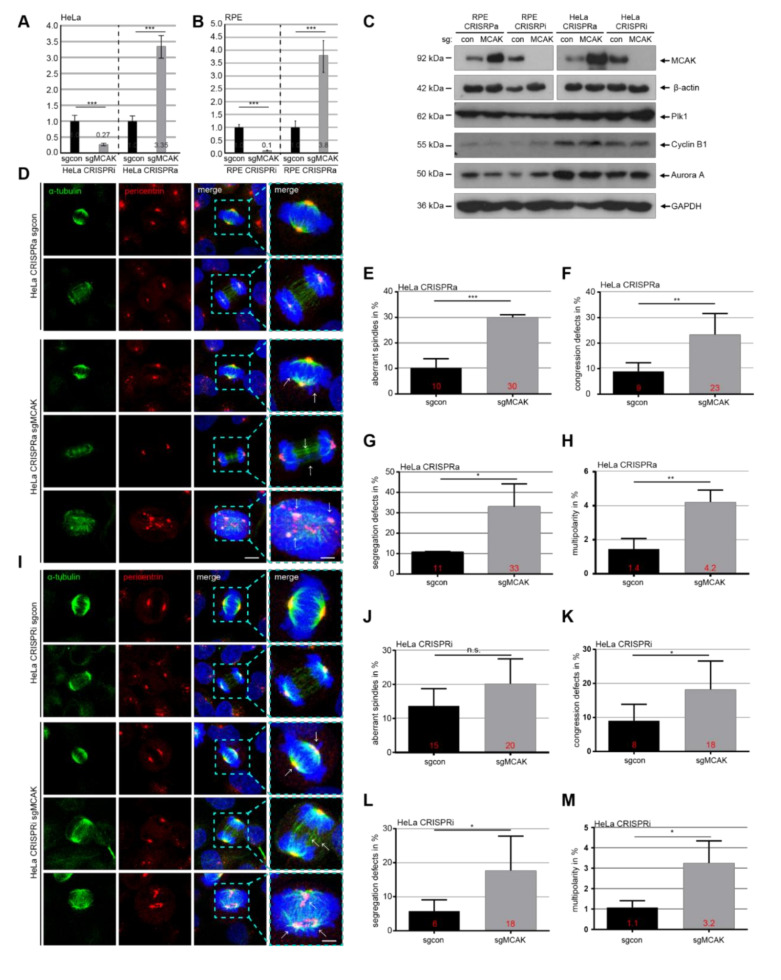
Mitotic defects in HeLa CRISPRi/a cells. (**A**,**B**) The gene level of *KIF2C* (MCAK) was analyzed in HeLa/RPE CRISPRi/a cells (i for inhibition/suppression, a for activation/overexpression, sgMCAK, targeting MCAK), compared to their corresponding control cells (sgcon). Glyceraldehyde-3-phosphate dehydrogenase (GAPDH) served as the endogenous control. The data are based on three independent experiments and presented as RQ with minimum and maximum range and statistically analyzed. RQ: relative quantification of the gene expression. (**C**) Control Western blot analysis. The membrane was stained with indicated antibodies and GAPDH served as the loading control. (**D**) HeLa CRISPRa cells were stained with antibodies against α-tubulin (green), pericentrin (red) and DAPI (4,6-diamidino-2-phenylindole) for confocal microscopy. Representatives are shown. White arrows indicate misaligned chromosomes, defective segregation or multipolar spindles. Scale: 7.5 μm, inset scale: 2.5 μm. (**E**–**H**) Evaluation of aberrant spindles (**E**), misaligned chromosomes (**F**), failed segregation (**G**), and multipolar spindles (**H**) in HeLa CRISPRa cells. (**I**) Representatives of HeLa CRISPRi cells are shown. White arrows indicate misaligned chromosomes, defective segregation or multipolar spindles. Scale: 7.5 μm, inset scale: 2.5 μm. (**J**–**M**) Evaluation of aberrant spindles (**J**), misaligned chromosomes (**K**), failed segregation (**L**), and multipolar spindles (**M**) in HeLa CRISPRi cells. Results presented in (**E–H**) and (**J–M**) are from three independent experiments (*n* = 3, 50 mitotic cells for each condition in each experiment) and presented as mean ± SEM. Student’s *t*-test was used. * *p* < 0.05, ** *p* < 0.01, *** *p* <  0.001. Abbreviation: n.s.: not significant.

To investigate the role of MCAK in regulating cell migration and motility, we first generated HeLa (cervix carcinoma cells) and RPE (retina epithelial cells) cell lines knockdown or overexpression of MCAK, utilizing a novel CRISPR-associated catalytically inactive dCas9 system with RNA-guided protein targeting [30]. To confirm the functionality and efficiency of the system, MCAK’s gene and protein level were checked in CRISPRi/a cells stably transfected with sgRNA against MCAK (sgMCAK) or a control sequence (sgcon). The relative gene expression of *KIF2C* was reduced by 73% in HeLa CRISPRi sgMCAK (HeLa CRISPRi) cells and by 90% in RPE CRISPRi sgMCAK (RPE CRISPRi) cells compared to both control sgcon cells (sgcon) (Figure 1A,B, 1st and 2nd bars). Likewise, HeLa CRISPRa sgMCAK (HeLa CRISPRa) and RPE CRISPRa sgMCAK (RPE CRISPRa) cells displayed an increased *KIF2C* gene expression by 236% and 281%, respectively (Figure 1A,B, 3rd and 4th bar). Further Western blot analyses underscored the efficient knockdown and overexpression of MCAK in CRISPRi/a cells (Figure 1C, 1st lane). Motility and migration of cells is coupled to and dependent on the cell cycle [31], and MCAK is an important MT-depolymerase necessary for mitotic progression [16,32]. To exclude the possibility that changed cell motility and migration could be caused by an altered cell cycle progression, we first analyzed the cell cycle distribution of the cell lines. The protein levels of Aurora A, cyclin B1 and Plk1 (Polo-like kinase 1), important cell cycle regulators, were hardly changed by the knockdown or overexpression of MCAK in CRISPRi/a cells compared to their respective control sgcon cell lines (Figure 1C, 2nd to 4th lanes). Cell viability assay and cell cycle distribution analysis demonstrated no significant alterations between the cell lines (Figure S1A,B). These findings are in contrast to two recent publications in thyroid carcinoma and hepatocarcinoma cells, which showed that a knockdown of MCAK leads to a reduced proliferation rate in these cell types [33,34]. This contradiction might be explained because both publications used a transient siRNA transfection system for their experiments or due to a cell type specific effect. These data suggest that knockdown or overexpression of MCAK has no significant effect on the cell cycle distribution and cell proliferation of HeLa/RPE CRISPRi/a cells.

### 3.2. Interfering with the Expression of MCAK Triggers Mitotic Defects

Since MCAK is involved in the mitotic progression by modulating the MT dynamics [16,32], we asked if its knockdown or overexpression induced mitotic defects in CRISPRi/a cells, as reported [16,18]. HeLa and RPE CRISPRi/a cells were stained for MT marker α-tubulin, centrosome marker pericentrin and DNA to microscopically analyze their mitotic phenotype. Indeed, 30% of HeLa CRISPRa cells, compared to 10% of HeLa sgcon cells, showed short mitotic spindles, less nucleated and thin MTs (Figure 1D,E, 3rd to 5th panel), a characteristic phenotype for hyperactive MCAK [24]. Additionally, 20% of HeLa CRISPRi cells exhibited aberrant spindles with long MTs and disorganized astral MTs (Figure 1I,J, 3rd to 5th panel), though not significant. In accordance with the defective spindle morphology, both HeLa CRISPRi and CRISPRa cells displayed an increased defective chromosome congression by 23% and 18%, respectively, compared to corresponding control sgcon cells in metaphase (Figure 1D,F,I,K, 3rd panels). Both HeLa CRISPRi/a cells also had deficiencies in chromosome segregation in anaphase (33% of CRISPRa vs. 11% of sgcon; 18% of CRISPRi vs. 6% of sgcon) (Figure 1D,G,I,L, 4th panels). The rates of multipolar cells with centrosome fragmentation were at a low level in both HeLa CRISPRa or CRISPRi cells, yet slightly elevated compared to respective control sgcon cells (4.2% of CRISPRa vs. 1.4% of sgcon; 3.2% of CRISPRi vs. 1.1% of sgcon) (Figure 1D,H,I,M, 5th panels).

RPE CRISPRi/a cells showed similar effects with significantly increased rates of aberrant spindles, defective chromosome congression, as well as segregation (Figure S1C,D). However, compared to HeLa cells, the extent of these mitotic defects was clearly reduced in RPE cells, likely because of their benign origin with a functional spindle assembly checkpoint and an efficient mitotic error correction capacity. Nevertheless, these data convey the notion that finely tuned control of MCAK is vital for faithful mitotic progression, and its overexpression or downregulation compromises the regulation of dynamic MTs, resulting in defective mitosis, as reported [16,35]. Besides, these results highlight that the CRISPR/dCas9 system works efficiently and is useful for functional analysis of targeted genes [16,18,32].

### 3.3. MCAK Overexpression or Downregulation Impairs Cell Motility and Migration

**Figure 2:**
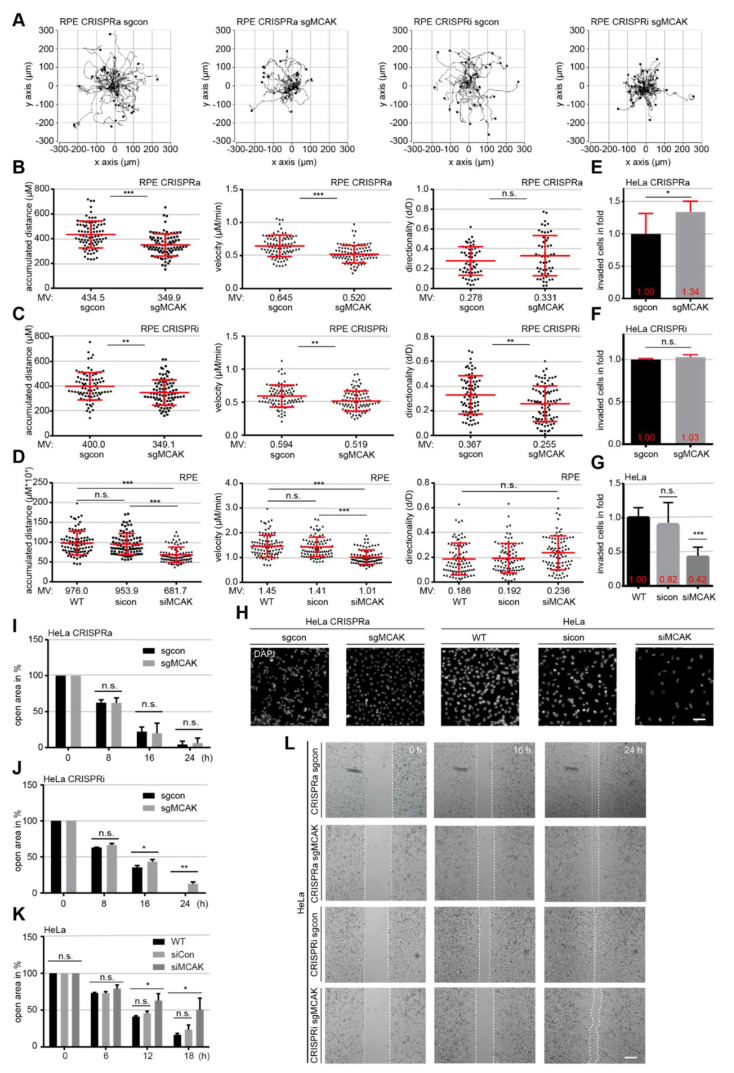
Down-regulated or up-regulated MCAK leads to reduced motility and migration rates. (**A**–**D**) Time-lapse microscopy was performed with RPE CRISPRi/a cells and RPE cells transiently depleted of MCAK (RPE siRNA). Random motility of these cells was analyzed. Representative trajectories of individual cells (*n* = 30) are shown (**A**). RPE CRISPRa (**B**), RPE CRISPRi (**C**), and RPE siRNA cells (**D**) were evaluated for accumulated distance (1st), velocity (2nd), and directionality (3rd), and the results are from three independent experiments, shown as scatter plots with variations. (**E**–**H**) Invasion assays. The total numbers of invasive HeLa sgcon cells for CRISPRi (**E**) and for CRISPRa (**F**) or HeLa cells (**G**) were assigned as 100%. The results are from three independent experiments and presented as mean ± SEM. (**H**) Representatives of invaded HeLa CRISPRa or HeLa siRNA cells are shown. Scale: 25 μm. (**I**–**L**) Wound healing/migration assays were performed with HeLa CRISPRi/a (**I**,**J**) or HeLa siRNA cells (**K**), and images were taken at indicated time points to document the migration front. (**I**–**K**) Quantification of the open area between both migration fronts at various time points. The cell-free area at 0 h was assigned as 100%. The results are from three independent experiments and presented as mean ± SEM. (**L**) Representatives are shown. White dashed line depicts the migration front. Scale: 300 μm. Unpaired Mann–Whitney *U* test was used in (**B–D**). ** *p* < 0.01, *** *p* < 0.001. Student’s *t*-test was used in (**E–G**,**I**,**J**,**K**). * *p* < 0.05, ** *p* < 0.01, *** *p* < 0.001. Abbreviation: n.s.: not significant.

It is well established that properly regulated MT dynamics is required for cell motility and migration, by regulating intracellular transport, and recycling of adhesion proteins and other important FA structure proteins [5,36]. To examine the impact of MCAK on cellular motility, RPE or HeLa CRISPRi/a cells were tracked using time-lapse microscopy for evaluating the accumulated distance, velocity and directionality of single tracked cells, as reported [37]. Compared to control sgcon cells, RPE CRISPRa cells showed a reduction of 19.5% in accumulated distance (435 vs. 350 µm) and velocity (0.65 vs. 0.52 µm/s) (Figure 2A,B, 1st and 2nd trajectories). A decrease of 12.8% in accumulated distance (400 vs. 349 µm) and velocity (0.59 vs. 0.51 µm/s) was observed in RPE CRISPRi cells compared to their control cells (Figure 2A,C, 3rd and 4th trajectories). Interestingly, HeLa CRISPRa cells did not present any significant changes (Figure S2A,C, 1st and 2nd trajectories), whereas HeLa CRISPRi cells reduced their accumulated distance and velocity in comparison with sgcon control cells (Figure S2B,C, 3rd and 4th trajectories). To exclude possible off-target effects of the CRISPR/dCas9 system and to corroborate these data, HeLa and RPE cells were transiently transfected with siRNA against MCAK (Figure S3A,C,J) for time-lapse microscopy. These cells presented a significantly reduced accumulated distance and velocity compared to cells treated with control siRNA (Figure 2D, Figures S2A–C and S3A,B,F). Furthermore, comparable results were also observed in malignant breast cancer MDA-MB 231 cells depleted of MCAK (Figure S3C–E). Directionality, an important parameter for directed cell migration, showed only minor changes in all analyzed cell types, likely owing to the lack of an attractant, which is absent in this experimental setup (Figure 2B–D, Figures S2A,B and S3E,F, 3rd scatter plots).

Cellular motility is a prerequisite for migration and invasion processes, and MCAK has recently been described as a metastatic factor for hepatocellular and nasopharyngeal carcinoma [38,39]. To address this issue, we analyzed the impact of MCAK overexpression or downregulation in an invasion assay, where cells have to invade through a Matrigel layer. HeLa CRISPRa cells displayed an increased invasion rate of 34% compared to sgcon cells, whereas CRISPRi cells did not show significantly different invasion rate vs. their control cells (Figure 2E,F,H, 1st and 2nd panel), despite their reduced motility (Figure S2B). Interestingly, HeLa cells transiently depleted of MCAK with siRNA demonstrated a significant decrease of 48.8% in invasion compared to HeLa sicon cells (Figure 2G,H, 3rd to 5th panel), suggesting a possible compensatory mechanism in HeLa CRISPRi cells through the stable long-term knockdown of MCAK.

Finally, we investigated the targeted migration capability using a wound-healing assay in HeLa CRISPRi/a cells. These cells considerably decreased their migration rate, especially at the late time-points (Figure 2I,J,L). To corroborate these results, HeLa and MDA-MB-231 cells were transiently depleted of MCAK and subjected to a wound-healing assay. Again, cells with less MCAK showed a lower migration capacity compared to their control cells (Figure 2K and Figure S3G–J), which was also slower than HeLa CRISPRi cells (Figure 2J vs. Figure 2K). These findings strongly suggest that the precise regulated expression of MCAK is required for cell motility, and both overexpression and knockdown compromise this capability.

### 3.4. Interfering with the Expression of MCAK Impacts the Timely Accumulation of Paxillin, FAK, and Their Phosphorylated Forms in FAs

**Figure 3:**
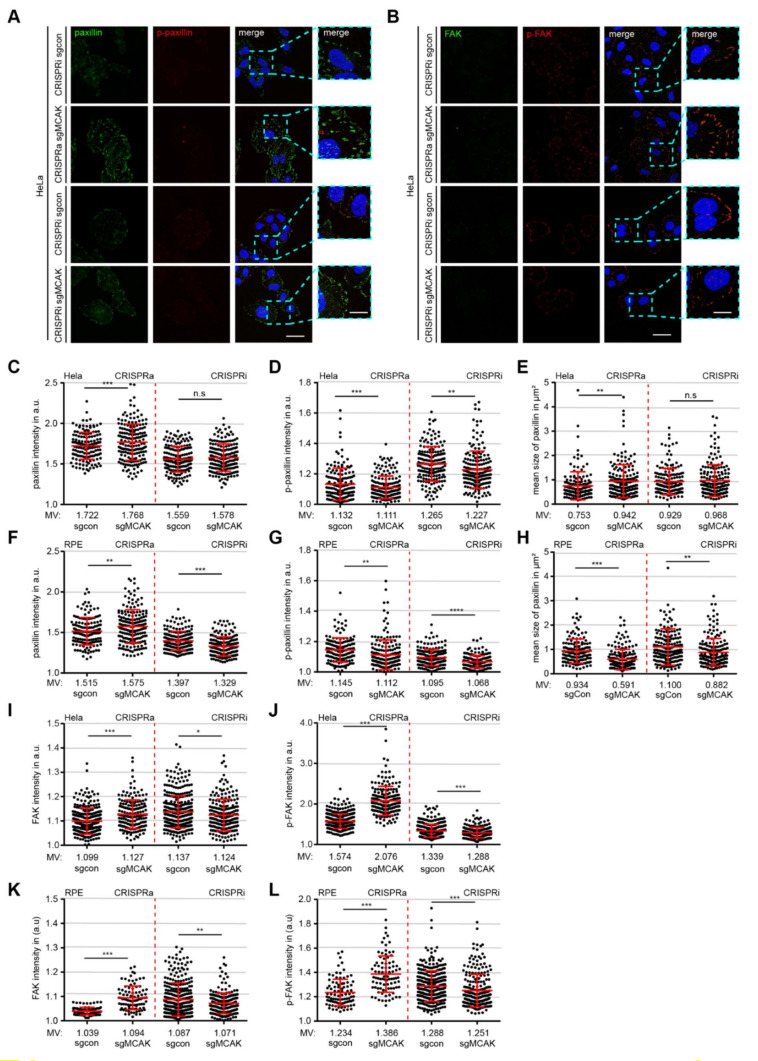
Interfering with the expression of MCAK deregulates FA proteins and their phosphorylation status (**A**,**B**) HeLa CRISPRi/a cells were stained for paxillin (green) and p-paxillin (red) (**A**), or FAK (green) and p-FAK (red) (**B**) for fluorescence microscopy. Representative images are shown. Scale: 12.5 µm; inset scale: 5 µm. (**C–H**) Quantification of the mean gray intensity of the outlined paxillin (**C**,**F**), p-paxillin (**D**,**G**) signals, and the signal size of paxillin (**E**,**H**) of HeLa and RPE CRISPRi/a cells (100 FAs measured in each experiment). The results are based on three independent experiments and presented as scatter plots showing mean ± SEM; a.u., arbitrary units. (**I–L**) Quantification of the mean fluorescence intensity of FAK (**I**,**K**) and p-FAK (**J**,**L**) signals of HeLa and RPE CRISPRi/a cells (100 FAs measured in each experiment). The results are based on three independent experiments and presented as scatter plots showing mean ± SEM. Unpaired Mann–Whitney *U* test was used. * *p* < 0.05, ** *p* < 0.01, and *** *p* < 0.001. Abbreviation: n.s.: not significant.

MTs are important for the interaction with FA components, for FA assembly as well as disassembly [5,40]. We were interested if MCAK, a modulator of MT dynamics, plays a role in the accumulation and phosphorylation of two crucial FA proteins, namely paxillin and FAK. To study this issue, RPE CRISPRi/a and HeLa CRISPRi/a cells were stained for paxillin, FAK and their phospho-forms (p-FAK: Y397, p-paxillin: T118). Both RPE CRISPRa and HeLa CRISPRa cells displayed a marginal but significant accumulation of paxillin in FAs compared to their respective sgcon cells (HeLa CRISPRa: 2.6%; RPE CRISPRa: 3.9%) (Figure 3A,C,F, 1st and 2nd scatter plots). While RPE CRISPRi cells reduced accumulation of these proteins in FAs could be observed in RPE CRISPRi cells by showing a reduced paxillin intensity of 4.8%, there was hardly a change observed in HeLa CRISPRi cells (Figure 3A,C,F, 3rd and 4th scatter plots). Strikingly, both HeLa CRISPRi/a and RPE CRISPRi/a cells displayed a significantly reduced mean intensity of phosphorylated paxillin (p-paxillin) (Figure 3A,D,G, 1st to 4th scatter plots), despite the increased paxillin intensity in CRISPRa cells.

The size of FAs is another important factor to predict the migration capacity of cells [41,42,43]. The size of the paxillin signal in FAs was measured and used as FA size. HeLa CRISPRa cells displayed a moderate increase in FA size, whereas no significant differences were observed between HeLa CRISPRi cells and their sgcon cells (Figure 3E, 1st to 4th scatter plots). By contrast, the non-tumorigenic RPE CRISPR cells reduced their FA size by 37% for CRISPRa and 19.8% for CRISPRi cells compared to their sgcon counterparts (Figure 3H, 1st to 4th scatter plots). In line with the paxillin data (Figure 3C,D,F,G), knockdown of MCAK in HeLa CRISPRi and RPE CRISPRi cells led to a significant decreased FAK and p-FAK intensity (Figure 3B,I–L, 3rd and 4th scatter plots). Surprisingly, like in RPE CRISPRa cells (Figure 3K,L, 1st and 2nd scatter plots), HeLa CRISPRa cells showed an increased signal intensity of FAK and p-FAK in FAs (Figure 3B,I,J, 1st and 2nd scatter plots), which might explain the improved invasion capacity observed in HeLa CRISPRa cells (Figure 2E). Additionally, the experiments were performed with MDA-MB-231 and HeLa cells transiently transfected with siRNA against MCAK (Figure S4). The transient knockdown of MCAK in both cell types induced a similar phenotype as observed in CRISPRi cells, with reduced paxillin, p-paxillin, FAK, and p-FAK intensity in FAs, and a decreased FA size (Figure S4). These results reinforce the notion that a balanced MCAK expression is required for a proper FA protein composition and their accurate phosphorylation status in space and time.

### 3.5. Deregulation of MCAK Delays CELL Spreading and Adhesion

**Figure 4:**
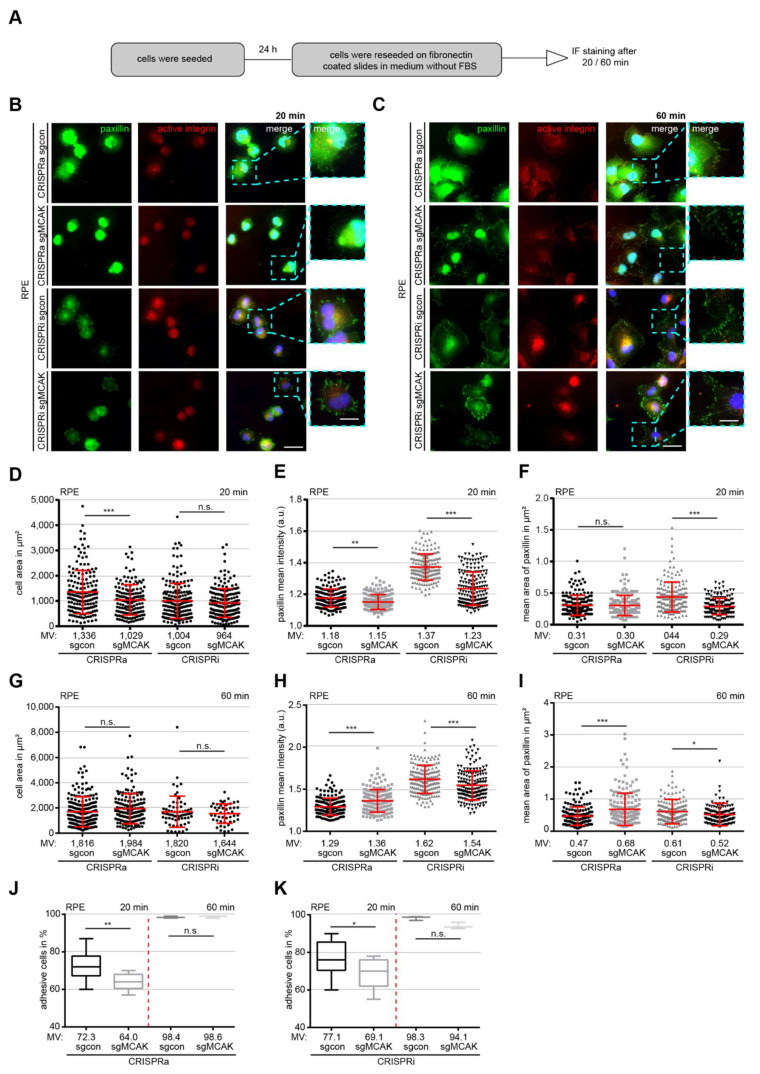
Deregulation of MCAK impairs the cell spreading and adhesion capacity**.** (**A**) Schedule of cell spreading/adhesion assay. (**B**,**C**) RPE CRISPRi/a cells were stained for active integrin (red), paxillin (green), and DNA (DAPI, blue). Representatives of 20 min and 60 min cell re-adhesion are shown. Scale: 25 µm; inset scale: 10 µm. (**D**,**G**) Quantification of cell size of RPE CRISPRi/a sgcon and sgMCAK cells at 20 min (**D**) and 60 min (**G**) after reseeding. The results are based on three independent experiments and presented as scatter plots showing mean ± SEM (180 cells). (**E**,**H**) Quantification of the mean fluorescence intensity of paxillin after 20 min (**E**) and 60 min (**H**) after reseeding (180 FAs measured in each experiment). The results are based on three independent experiments and presented as scatter plots showing mean ± SEM. (**F**,**I**) Measurement of the paxillin signal size after the cells were reseeded for 20 min (**F**) and 60 min (**I**) (at least 60 FAs measured in each experiment). The results are based on three independent experiments and presented as scatter plots showing mean ± SEM. (**J**,**K**) Quantified percentage of fully re-attached cells after 20 min (**J**) and 60 min (**K**) (50 cells measured in each experiment). The results are based on three independent experiments and presented as box plots showing mean ± SEM. Unpaired Mann–Whitney *U* test for (**D**–**I**). Student’s *t*-test for (**J**,**K**). * *p* < 0.05, ** *p* < 0.01, and *** *p* < 0.001. Abbreviation: n.s.: not significant.

To examine the cell spreading and adhesion capacity, which is highly dependent on the timely accumulation and phosphorylation of FA components, RPE CRISPRi/a cells were subjected to a re-adhesion assay [43,44]. The cells were trypsinized, reseeded on fibronectin-coated slides for 20 or 60 min in medium containing FBS (Figure 4A), stained for active integrin, a marker for adherent cells, and paxillin, and microscopically evaluated for cell size, paxillin FA size and the paxillin mean intensity (Figure 4B–K). The cell size was significantly decreased only in RPE CRISPRa cells after 20 min with a reduction of 23% compared to sgcon cells (Figure 4B,D,G). Interestingly, both RPE CRISPRa and RPE CRISPRi cells displayed a reduced paxillin intensity (Figure 4E), paxillin signal area (Figure 4F) and less adhesive cells (Figure 4J,K, 1st and 2nd scatter plots) in comparison to their sgcon cells (Figure 4E,F,J,K). After 60 min, both the cell size and number of adherent cells were comparable to the levels of RPE CRISPRi/a control cells (Figure 4G,J,K). The paxillin intensity was also similar to that under undisturbed culture conditions (Figure 3F vs. Figure 4H). Surprisingly, the paxillin area was increased by 45% in RPE CRISPRa cells (Figure 4I, 1st and 2nd scatter plots), which might be explained by a delayed FA disassembly caused by MCAK’s hyperactive MT depolymerization activity.

Comparable results were also obtained with HeLa CRISPRi/a cells (Figure S5A–H). Especially, after 20 min, both the cell size and the percentage of adherent cells were significantly changed (Figure S5A,G,H, 1st and 2nd scatter plots), indicating a decreased cell spreading capacity of cells with deregulated MCAK expression. These data suggest that overexpression or knockdown of MCAK compromises FA formation, and inhibits cell spreading and adhesion, especially at early time points.

### 3.6. MCAK Modulates Actin- and MT Dynamics

**Figure 5:**
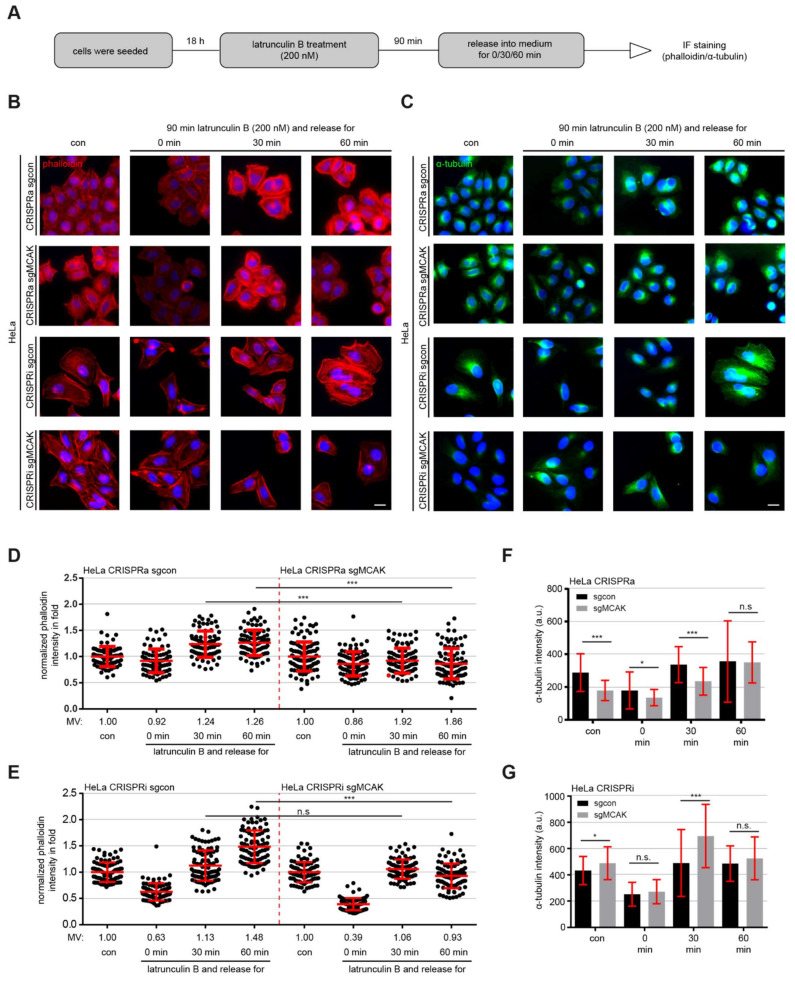
Deficient actin and MT repolymerization after latrunculin B treatment in MCAK overexpressing or knockdown cells**.** (**A**) Schedule of latrunculin B washout experiment. HeLa CRISPRi/a cells were treated with 200 nM latrunculin B for 90 min and released into fresh medium and incubated for indicated time periods. (**B**,**C**) The cells were stained for F-actin (phalloidin, red) (**B**) and α-tubulin (green) (**C**). Representatives of actin fiber and MT reassembly are shown. Scale: 25 µm. (**D**,**E**) Quantification of the mean fluorescence intensity of F-actin (phalloidin) per cell (30 cells per condition) in HeLa CRISPRa (**D**) and CRISPRi (**E**) cells. The results are based on three independent experiments and presented as scatter plots showing mean ± SEM. (**F**,**G**) Quantification of the MT repolymerization by measuring the mean fluorescence intensity of α-tubulin (30 cells per condition) in HeLa CRISPRa (**F**) and CRISPRi (**G**) cells. The results are based on three independent experiments and presented as bar graphs showing mean ± SEM. Unpaired Mann–Whitney *U* test for (**D**,**E**). Student’s *t*-test for (**F**,**G**). * *p* < 0.05 and *** *p* < 0.001. Abbreviation: n.s.: not significant.

The intensive crosstalk between the actin- and MT cytoskeleton is fundamental for their orchestrated dynamics and proper cell migration [4]. To analyze if MCAK has an impact on this actin-MT relationship, HeLa CRISPRi/a cells were treated with latrunculin B, an actin monomer-sequestering agent that blocks fast actin polymerization and abolishes stress fiber formation, leading to a reversible disruption of the actin cytoskeleton [45]. Cells were treated with latrunculin B for 90 min and released in fresh medium for 0, 30 and 60 min, and stained for F-actin with phalloidin and α-tubulin (Figure 5A). By comparing the repolymerization rate of F-actin, both HeLa CRISPR sgcon cell types showed an efficient actin repolymerization (Figure 5B,D,E, 1st to 4th scatter plots). After 30 and 60 min, both cell lines demonstrated higher F-actin levels than untreated cells, which was due to synchronized repolymerization of actin filaments (Figure 5B–E, 1st to 4th scatter plots). In contrast, HeLa CRISPRa cells were unable and HeLa CRISPRi cells were just slightly able to repolymerize their F-actin content to their basal level even after 60 min of release (Figure 5B–E, 5th to 8th scatter plots). Comparable results were also observed with RPE CRISPRi/a cells (Figure S6A,B).

Despite latrunculin B being described as an agent disrupting the actin cytoskeleton without affecting the MTs at low concentrations (30 nM) [46], we analyzed its impact on the MT dynamics during the same timeframe of both HeLa CRISPRi/a and RPE CRISPRi/a cells treated with 200 nM of latrunculin B used in the experiments (Figure 5). Interestingly, the α-tubulin content of control cells without latrunculin B treatment was reversely correlated with the depolymerization activity of overexpressed or knockdown of MCAK (Figure 5F,G and Figure S6C,D). Both HeLa and RPE CRISPRa cell lines displayed a significantly reduced α-tubulin intensity in IF staining and decreased, but not significant, α-tubulin levels in flow cytometry (FACS) analyses as well (Figure 5F and Figure S6C,D,F). In further support, knockdown of MCAK led to an increased MT intensity in both assays (Figure 5G and Figure S5D–F). More importantly, both cell lines treated with 200 nM of latrunculin B impaired MT nucleation by showing faint and depolymerized MTs (Figure 5C,F,G, 1st vs. 2nd IF panels). After 30 min release, CRISPRa cells clearly decreased the repolymerization rate of MTs (Figure 5F and Figure S6C, 5th and 6th bar graph), whereas CRISPRi cells highly enhanced this rate (Figure 5G and Figure S6D, 5th and 6th bar graph). After 60 min release, the cells started to equalize their α-tubulin content in HeLa CRISPRi/a cells, though with big standard derivations (Figure 5F,G, 7th and 8th graph). These data could explain the impaired actin dynamics observed in CRISPRi/a cells released from latrunculin B treatment (Figure 5B,D,E), since the actin- and MT-network are functionally coupled by various cross-linking proteins, and MT organization can restrict F-actin alignment [47,48], further enhancing the indirect regulation of cell motility by MCAK.

### 3.7. Overexpression or Suppression of MCAK Perturbs MT-Induced FA Turnover

**Figure 6:**
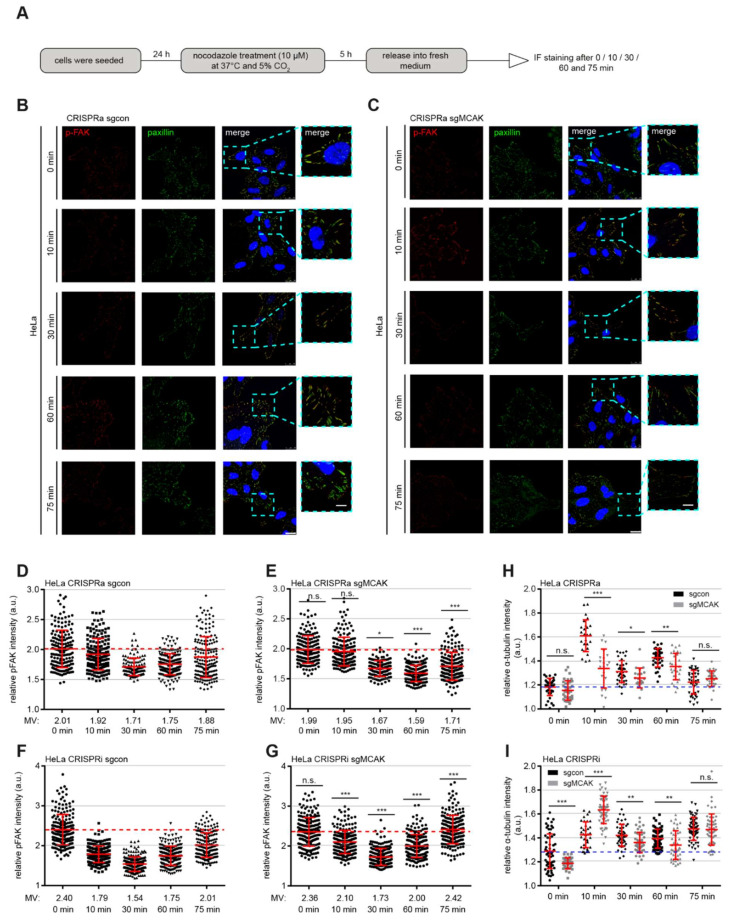
CRISPRi/a cells display a delayed MT-induced FA disassembly and assembly. (**A**) Schedule of nocodazole washout experiment. HeLa CRISPRi/a cells were treated with 10 µM nocodazole for 5 h and released into fresh medium for indicated time points. (**B**,**C**) HeLa CRISPRa sgcon (**B**) and CRISPRa sgMCAK (**C**) cells were stained for p-FAK (red), and paxillin (green). Representatives of FA disassembly and assembly are shown. Scale: 25 µm, inset scale: 12.5 µm. (**D–G**) Quantification of the mean fluorescence intensity of p-FAK (red) per FA (90 FAs per condition and experiment) in HeLa CRISPRa (**D**,**E**) and CRISPRi (**F**,**G**) cells. The results are based on three independent experiments and presented as scatter plots showing mean ± SEM, a.u., arbitrary units. (**H**,**I**) Quantification of the MT repolymerization by measuring the mean fluorescence intensity of α-tubulin in whole cells (30 cells per condition) in HeLa CRISPRa (**H**) and CRISPRi (**I**) cells. The results are based on three independent experiments and presented as bar graphs showing mean ± SEM. Unpaired Mann–Whitney *U* test was used. * *p* < 0.05, ** *p* < 0.01, and *** *p* < 0.001. Abbreviation: n.s.: not significant.

A balanced assembly and disassembly of FAs is the key for determining cell motility and migration rate [42]. Impaired actin- as well as MT dynamics, together with altered paxillin/FAK accumulation and phosphorylation, argue for an impaired FA turnover. To analyze this aspect in detail, a well-established washout assay [43,49] was used, which is based on the treatment with nocodazole, a MT depolymerizing agent [50]. The washout of nocodazole immediately triggers the MT repolymerization, leading first to a coordinated FA disassembly, and subsequently to a new FA assembly [49]. This allows the analysis of MCAK’s role in the process of FA turnover. HeLa CRISPRi/a cells were treated with nocodazole and then released for 0, 10, 20, 30, 60 and 75 min. Cells were stained for p-FAK and paxillin, two important FA assembly factors [43,51], and α-tubulin for microscopic evaluation (Figure 6A). After nocodazole washout, both HeLa CRISPRa and their control cells started to disassemble their FAs indicated by reduced p-FAK signals from the nocodazol treatment to 30 min of the release (Figure 6B–E). However, HeLa CRISPRa cells required 60 min to completely disassemble their FAs (Figure 6E, 4th scatter plot), when their control sgcon cells already started to assemble their FAs again (Figure 6D, 4th scatter plot). The significant difference in the p-FAK intensity between HeLa CRISPRa and their control cells was still observable after 75 min of nocodazole release (Figure 6D,E, 5th scatter plots), suggesting a delayed FA turnover rate in HeLa CRISPRa cells overexpressing MCAK. By contrast, HeLa CRISPRi cells showed an impaired disassembly of their FAs after 30 min of nocodazole washout (0 min: 2.36; 30 min: 1.73 a.u.) compared to sgcon cells (0 min: 2.40; 30 min: 1.54 a.u.) (Figure 6F,G, 1st to 3rd scatter plots). The p-FAK signal peaked at 75 min in CRISPRi cells (2.42 a.u.), whereas their counterpart sgcon cells still assembled their FAs (2.01 a.u.) (Figure 6F,G, 4th and 5th scatter plots). These results suggest that reduced MCAK expression leads to a MT-related disorganized disassembly and reassembly of FAs.

To underscore the role of the MT cytoskeleton during nocodazole release, the α-tubulin intensity was also measured. The depolymerization of the MT cytoskeleton by nocodazole possibly interferes with various tubulin post modifications like acetylation, glutamylation, tyrosination, glycosylation, and phosphorylation, which might affect the outcome of this experiment [52]. Both HeLa CRISPRi/a cells showed significantly perturbed repolymerization of MTs throughout the nocodazole release course (Figure 6H,I and Figure S6E). In particular, this was most significant at 10 min post-release time point, when all the MTs rapidly re-polymerized. HeLa CRISPRa displayed a dramatically reduced repolymerization rate (Figure 6H, 3rd and 4th scatter plots), whereas HeLa CRISPRi showed a massive increase in the α-tubulin intensity (Figure 6I, 3rd and 4th scatter plots). These results point to the notion that properly regulated MCAK is required for a dynamic MT cytoskeleton and a coordinated FA turnover.

### 3.8. MCAK Is Involved in FA Lifetime and MT Plus-Tip Dynamics

**Figure 7:**
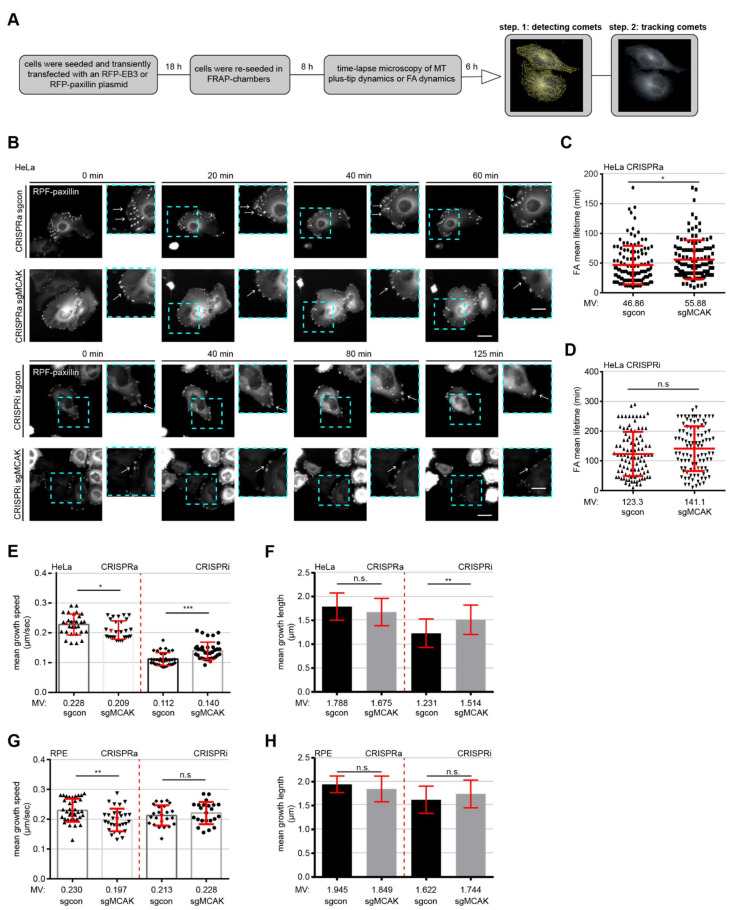
MCAK modulates FA stability and MT dynamics**.** (**A**) Schedule of the FA stability and EB3 comet tracing assay. Time-lapse microscopy was performed with HeLa CRISPRi/a cells transfected with RFP-paxillin (**B–D**) or RFP-EB3 (**E**–**H**). The analysis of the FA stability was conducted with ImageJ and the comet detection/tracking was executed with U-track Master, a MatLab plugin. (**B**) Representatives of the FA stability are shown in HeLa CRISPRi/a cells at indicated time points. Scale: 25 µm, inset scale: 12.5 µm. The mean FA lifetime (180 FAs per condition) is quantified in HeLa CRISPRa (**C**) and CRISPRi (**D**) cells. The results are based on three independent experiments and presented as scatter plots showing mean ± SEM. (**E**,**G**) Plus-end mean growth velocity of RFP-EB3 comets was evaluated in HeLa (**E**) and RPE (**G**) CRISPRi/a cells. The results are based on three independent experiments and presented as scatter plots showing mean ± SEM (30 cells per conditions, >45.000 comets tracked). (**F**,**H**) Mean RFP-EB3 comet track growth length was evaluated in HeLa (**F**) and RPE (**H**) CRISPRi/a cells. The results are based on three independent experiments and presented as bar graphs showing mean ± SEM (30 cells per conditions). Unpaired Mann–Whitney *U* test was used. * *p* < 0.05, ** *p* < 0.01, and *** *p* < 0.001. Abbreviation: n.s.: not significant.

To study the potential effect of MCAK on the FA lifetime in a more physiological condition, HeLa CRISPRi/a cells were transfected with RFP-paxillin to visualize FAs, re-seeded in FRAP (fluorescence recovery after photobleaching)-chambers, and time-lapse videos were taken for up to six hours for microscopic evaluation (Figure 7A). The RFP-paxillin fluorescence signal on single FA was measured until it decreased to the background level. Both HeLa CRISPRi and CRISPRa cells displayed an impaired FA disassembly illustrated by an increased FA mean lifetime (Figure 7B–D). The FA disassembly of HeLa CRISPRa cells was 19.25% slower compared to their sgcon cells (Figure 7B,C). A similar but not significant effect was observed in HeLa CRISPRi cells, which required 14.44% longer for a single FA turnover cycle in comparison to control cells (Figure 7B,D). These results further support the notion that interfering with the expression of MCAK leads to disrupted FA dynamics, leading to the compromised migration and motility of CRISPRi/a cells.

The MT plus-tip dynamics is an important parameter for cell motility, since it contributes to a front-rear cell polarity and coordinates FA turnover, promoting a robust directional migration [21,40]. To substantiate the regulatory role of MCAK on the MT plus-tip protein dynamics, a well-established EB3 (end-binding protein 3) comet detection assay [53] was performed with RPE and HeLa CRISPRi/a cells. The cells were transfected with RFP-EB3, a MT plus-tip tracking protein, re-seeded in FRAP-chambers, and time-lapse microscopy was performed up to five minutes with a camera speed at maximum velocity. Compared to their sgcon cells, the mean growth speed of the MT tracks was significantly reduced in HeLa CRISPRa and RPE CRISPRa cell lines by 8.33% and 14.35%, respectively (Figure 7E,G, 1st and 2nd scatter plots). Consistent with these observations, downregulation of MCAK resulted in increased MT growth speed, which was more pronounced in HeLa cells with an increase of 25% compared to RPE cells with 7.04% relative to the corresponding controls (Figure 7E,G, 3rd and 4th scatter plots). The mean growth length followed the same pattern with a decrease in CRISPRa cells and an elevation in CRISPRi cells, though only HeLa CRISPRi cells showed a significant increase by 23% (Figure 7F,H). These data highlight MCAK’s function in regulating the MT plus-tip dynamics.

## 4. Discussion

MCAK has been shown to be associated with the migration capacity of endothelial, epithelial and cervical carcinoma cells [19,20,21] by regulating the polarization of MT growth and the FA turnover depending on RAC-1 [20,21]. In the present study, we utilized the CRISPR/dCas9 system [22] to further elucidate MCAK’s molecular role in the process of cell motility and migration of cancer, as well as benign cells. This is a topic of significance, since it is well known that MCAK is associated with the metastasis of various cancer entities such as non-small cell lung cancer, gliomas, gastric and breast cancer [54,55,56,57,58]. Interfering with the expression of MCAK by either overexpression or downregulation caused a decreased cell motility and migration capacity attributed to impaired FA assembly and disassembly associated with compromised MT- and actin-cytoskeleton dynamics.

Specifically, our results obtained by flow cytometry, re-adhesion, and nocodazole washout assays highlight that MCAK significantly alters the polymerized MT content and dynamics, which is critical for the disassembly of FAs [59,60]. Further, we show that MCAK regulates the velocity and growth length of EB3 proteins bound to the plus-tips of polymerizing MTs, demonstrating MCAK’s important role as MT modulator. Up-regulated KIF2A, another kinesin-13 family member, and depolymerase was shown to be associated with poor prognosis in patients with lung adenocarcinoma, breast cancer and gastric cancer by promoting tumor growth and migration [61,62,63]. Despite its close relationship with MCAK and functions on the mitotic spindle, KIF2A increases the migration rate of cancer cells via the AKT serine/threonine kinase 1 signaling, independent of its depolymerase activity [62], which could not be verified in MCAK overexpressing cells.

The knockdown of MCAK led to an impaired recruitment of FAK and paxillin, two positive key regulators for cell migration [64,65], leading to the reduced phosphorylation of these important FA structure proteins. These results are in accordance with a recently published paper by Zong et al., showing that MCAK knockdown interfered with directional movement, centrosome positioning and increased lifetime of FA’s in RPE cells [21]. Moreover, both HeLa and RPE knockdown cells displayed a decreased FA size, which is also a vital factor related to cell migration [41]. The assembly of stable FAs is initiated by the activation of integrin receptors. This leads to the recruitment of multiple proteins including FAK, paxillin, talin and vinculin [66]. Furthermore, to ensure a stable complex, paxillin is able to bind to MTs by its LIM2/LIM3 domains, suppressing the catastrophe rates of MTs in FA complexes [13]. However, MTs regulate integrin activity and the remodulation of adhesions by coordinating the transport processes for delivering important ECM modulating proteins like matrix metalloproteinases and proteins inducing integrin endocytosis like dynamin-2 [40,67]. In combination with these observations, our data indicate that hyperstabilized MTs, induced by downregulation of the MT depolymerase MCAK, cause defects in the recruitment or stability of FA structure proteins. In accordance with this notion, the knockdown of RBP-J interacting and tubulin-associated protein (RITA) was shown to impair the FA turnover, migration and invasion in breast cancer cells as well as fibroblasts by interacting with α-actinin, lipoma preferred partner (LPP) and further stabilizing the MT cytoskeleton [43]. By contrast, the overexpression of MCAK is associated with an increased MT catastrophe rate, which stabilizes and enlarges FAs with elevated paxillin, FAK and p-FAK content in cancer cells. This could be associated with an impaired clathrin-mediated endocytosis of FA elements, since the MT cytoskeleton is crucial for the removal of recycled integrin and FA components encapsulated into vesicles [68]. This assumption is further strengthened by the data gained from the FA turnover experiment utilizing the MT depolymerization drug nocodazole [69]. This experiment showed that MCAK knockdown cells (CRISPRi) displayed severe failure in FA disassembly, whereas MCAK overexpression cells (CRISPRa) demonstrated serious defects in FA assembly, which is in line with their perturbed MT dynamics. In further support, the proto-oncogenic C-Src (SRC) was shown to phosphorylate EB1 on Y247 in the proximity of FAs [70]. This phosphorylation led to an enhanced MT catastrophe rate, comparable to MCAK CRISPRa cells, modulating the cell motility of HUVEC cells [70].

Similar to the formation of stable FAs, the adhesion and cell spreading process is initiated by paxillin and FAK binding directly to activated integrins [71,72]. Another key player for this process is the actin-myosin network, which is partly coordinated by a feedback mechanism between the RhoGTPase family and the MT-cytoskeleton [8]. The MT repolymerization after nocodazole treatment was shown to stimulate TIAM2, which in turn activates RAC1 on the leading edge of skin papilloma cells [10], stimulating actin repolymerization and membrane protrusion [8,10]. This is in line with previous data, that the overexpression of RAC1 has a dominant effect over the activity of MCAK, indicating that it could act downstream or in parallel to MCAK in mediating cell motility and polarity [21]. We show here that interfering with regulated MT dynamics by deregulation of MCAK leads to impaired cell adhesion and cell spreading, especially in the initiation process of FAs. The delay in re-adhesion is probably caused by the perturbed recruitment of FA proteins to newly assembling FAs, as shown for paxillin. Another important factor is the disturbed actin stress fiber formation and MT repolymerization after re-seeding, observed in the latrunculin B assay, since the actin polymerization functions as main driving force for cell–cell adhesion in epithelial and endothelial cells or in the adhesion process to the extracellular matrix [73,74,75].

Finally, our results suggest that overexpression, as well as knockdown of MCAK, impacts the lifespan of single FAs during cell migration, as suggested by Zong et al. [21]. The disassembly of FAs requires a multitude of proteins transported to mature FAs, which depends highly on coordinated MT dynamics such as the proper recycling and transport of integrins packed in endosomes [67,76]. Based on these findings, we propose that MCAK may have an indirect function in regulating the transport of cargos along MTs important for FA assembly and disassembly.

## 5. Conclusions

**Figure 8:**
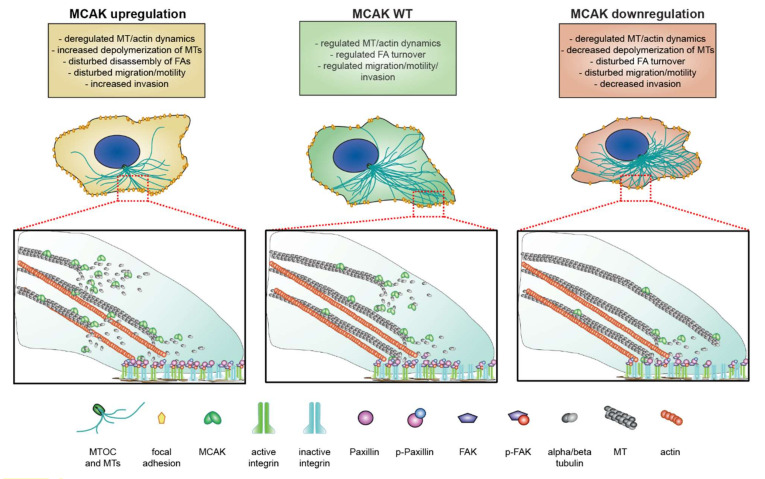
Deregulation of MCAK causes impaired motility and migration capacity, induced by altered MT dynamics and FA assembly as well as disassembly. Proposed working model of MCAK regulating FA assembly and disassembly by modulating the MT dynamics. Interfering with the expression of MCAK (overexpression: yellow; knockdown: red) leads to disturbed paxillin/p-paxillin/FAK/p-FAK levels in FAs. Moreover, the impaired MT dynamics are associated with delayed cell spreading and adhesion. The altered formation of stable FAs results in an uncoordinated assembly and disassembly of FAs during the migration/invasion process, compromising cell motility.

Our data suggest that a fine-tuned regulation of MCAK is important for cell motility and migration (Figure 8). Both overexpression and downregulation of MCAK causes an altered FA protein composition and activity, disturbed FA assembly and disassembly, delayed cell adhesion, and a less dynamic MT and actin network, leading to impaired cell motility and migration. Further studies are required to investigate the role of MCAK/MT plus-tip dynamics on endocytosis, and the anterograde/retrograde cargo transport involved in the assembly and disassembly of FAs.

## Data Availability

The data presented in this study are available on request from the corresponding author.

## Supplementary Materials

The following are available online at https://www.mdpi.com/article/10.3390/cancers13225673/s1, Figure S1: Mitotic defects in RPE CRISPRi/a sgMCAK cells, Figure S2: HeLa CRISPRa/i cells display reduced motility, Figure S3: downregulation or overexpression of MCAK leads to reduced motility and migration, Figure S4: Suppression of MCAK by siRNA deregulates FA proteins and their phosphorylation status, Figure S5: Downregulation or upregulation of MCAK in HeLa CRISPRa/I cells impairs the cell spreading and adhesion capacity, Figure S6: Deficient actin fiber and microtubule repolymerization in RPE CRISPRa/i cells treated with latrunculin B. Additional file 1: original WBs Figure 1.
