# Supplementary material for: Mitotic Centromere-Associated Kinesin (MCAK/KIF2C) Regulates Cell Migration and Invasion by Modulating Microtubule Dynamics and Focal Adhesion Turnover
Source: Cancers (Basel). 2021 Nov 12;13(22):5673. doi: 10.3390/cancers13225673 (PMC8616312; doi:10.3390/cancers13225673)
Supplement: Supplementary file 1 [file cancers-13-05673-s001.zip › cancers-1456482-supplementary.pdf]

Supplemental Information:

Mitotic centromere-associated kinesin (MCAK/KIF2C) regulates cell migration and invasion by modulating microtubule dynamics and focal adhesion turnover

Ha Hyung Moon[1], Nina-Naomi Kreis[1], Alexandra Friemel[1], Susanne Roth[1],

Dorothea Schulte[2], Christine Solbach[1], Frank Louwen[1],

Juping Yuan[1] and Andreas Ritter[1]

1 Division of Obstetrics and Prenatal Medicine, Department of Gynecology and Obstetrics, University Hospital Frankfurt, J. W. Goethe-University, Theodor-Stern-Kai 7, D-60590 Frankfurt, Germany

2 Institute of Neurology, Edinger Institute, University Hospital Frankfurt, J.W. Goethe University, Frankfurt, Germany

* Correspondence: Andreas.Ritter@kgu.de

Running title: Mitotic centromere-associated kinesin (MCAK/KIF2C) in cell migration and motility

Corresponding Authors:

Dr. Andreas Ritter: Email: andreas.ritter@kgu.de; Tel.: +49 69 6301 83297

Division of Obstetrics and Prenatal Medicine, Department of Gynecology and Obstetrics, University Hospital Frankfurt, J. W. Goethe-University, Theodor-Stern-Kai 7, D-60590 Frankfurt, Germany

We declare no conflict of interest, both financial and personal.

Supplemental Figure Legends

Fig. S1

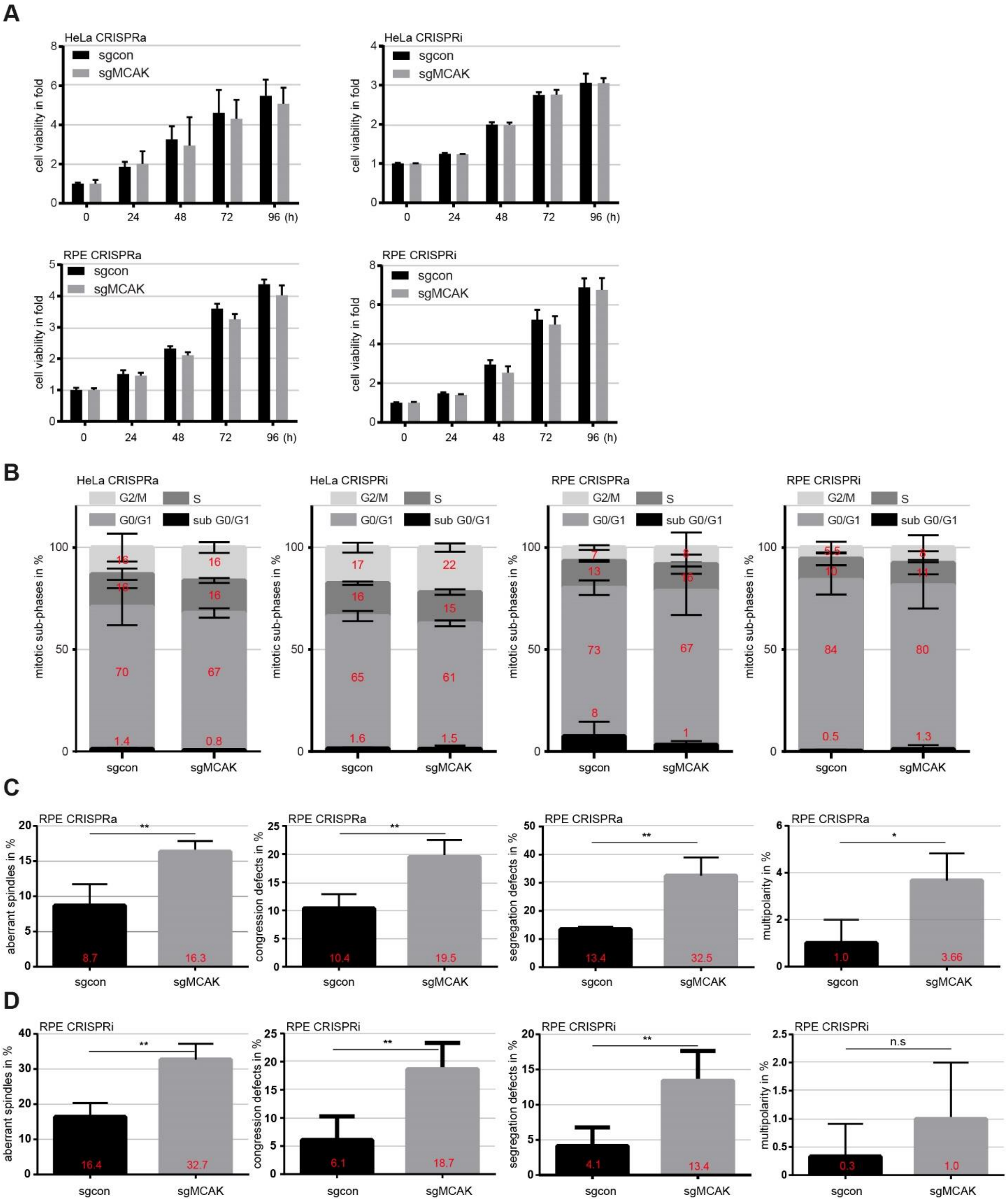

Figure S1: Mitotic defects in RPE CRISPRi/a sgMCAK cells

(**A**) Cell viability assay. HeLa and RPE CRISPRi/a cells (i for inhibition/suppression, a for activation/overexpression; sgMCAK, targeting MCAK; sgcon, control) were seeded in 96-well plates and cultured for 0, 24, 48, 72 and 96 h. Cell viability was measured. The results are from three independent experiments with HeLa CRISPRi/a cells and presented as mean ± SEM. Statistical analysis (Student's t-test) showed no significant difference. **(B)** Cell cycle distribution of HeLa CRISPRi/a and their control sgcon cells. The results are based on three independent experiments. **(C and D)** RPE CRISPRi/a cells were stained with antibodies against α-tubulin (green), pericentrin (red) and DAPI (4,6-diamidino-2-phenylindole) for confocal microscopy. (C) Evaluation of aberrant spindles (1st bar graph), misaligned chromosomes (2nd bar graph), failed segregation (3rd bar graph), and multipolar spindles (4th bar graph) in RPE CRISPRa cell lines. (D) Evaluation of aberrant spindles (1st bar graph), misaligned chromosomes (2nd bar graph), failed segregation (3rd bar graph), and multipolar spindles (4th bar graph) in RPE CRISPRi cells. Student's t-test was used. $*p < 0.05$, $**p < 0.01$, $***p < 0.001$.

Fig. S2

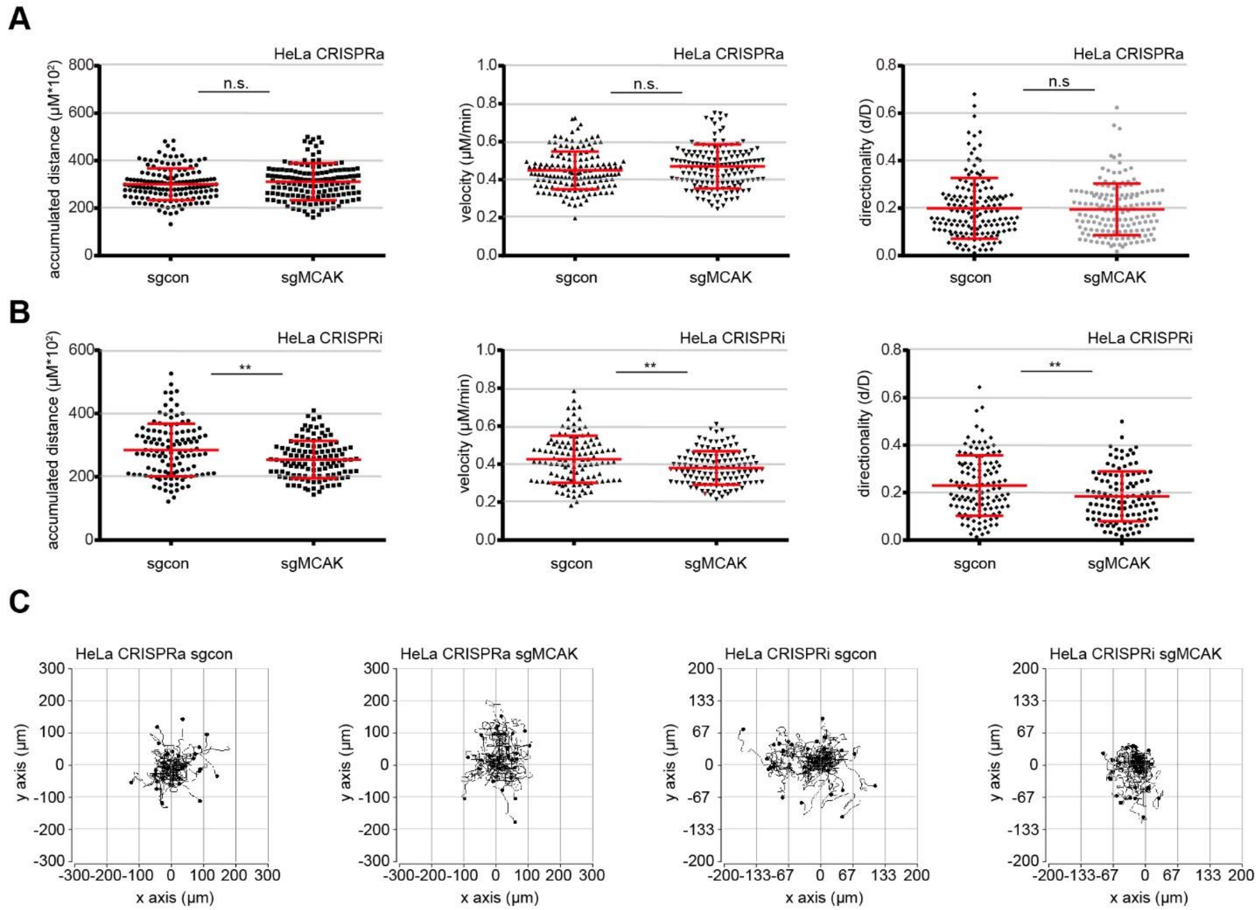

Figure S2: HeLa CRISPRa/i cells display reduced motility

(**A-C**) Time-lapse microscopy was performed with HeLa CRISPRi/a cells for up to 12 h. Random motility of these cells was analyzed. HeLa CRISPRa (A) and HeLa CRISPRi (B) were evaluated for accumulated distance (1st), velocity (2nd), and directionality (3rd). The results are from three independent experiments, and shown as scatter plots with variations. (C) Representative trajectories of individual cells (n = 30) are shown. Unpaired Mann-Whitney *U*-test was used in A and B. ∗∗p < 0.01, ∗∗∗p < 0.001.

Fig. S3

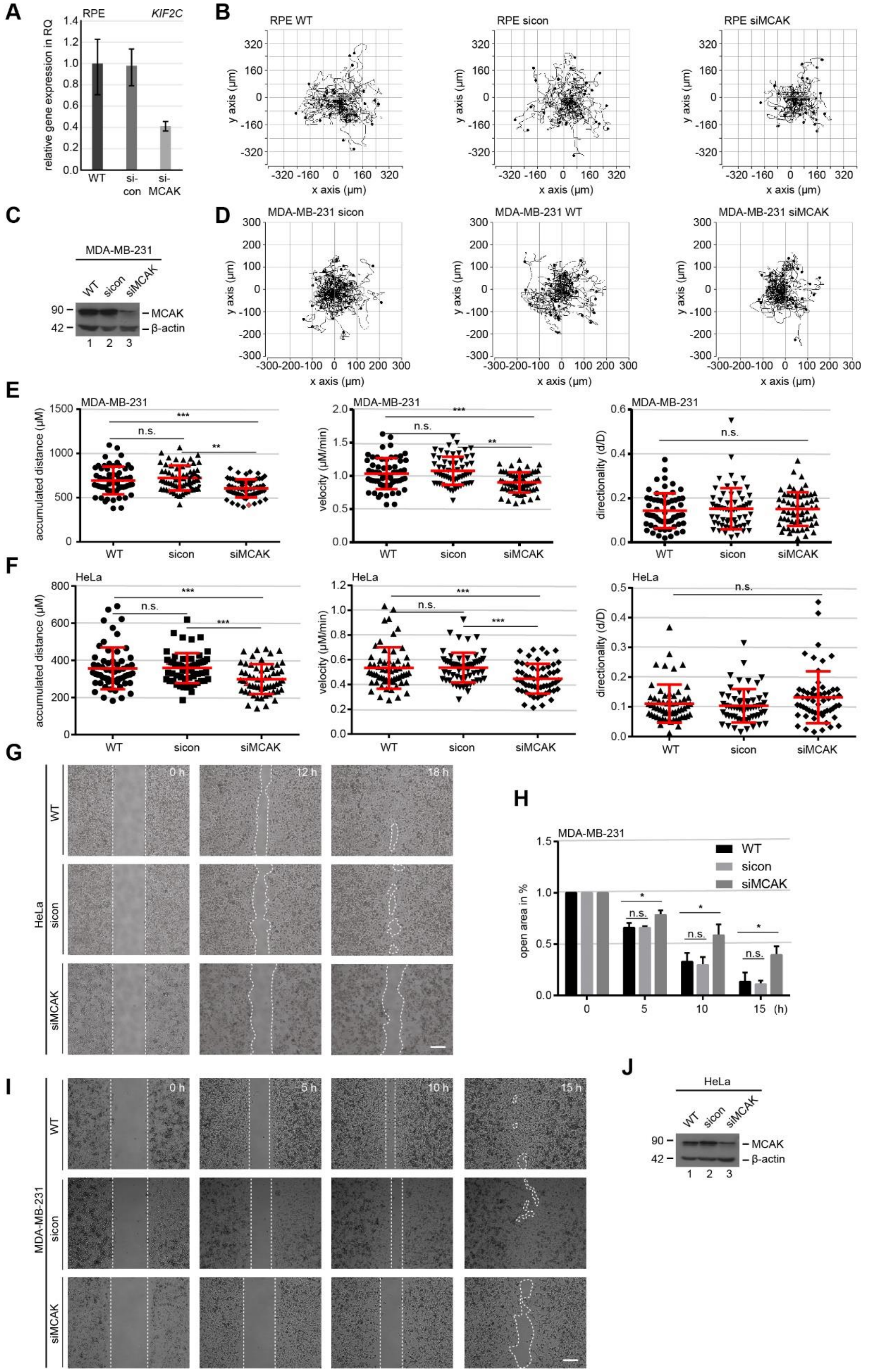
A
RPE
KIF2C
relative gene expression in RQ
WT
si-con
si-MCAK
B
RPE WT
RPE sicon
RPE siMCAK
x axis (µm)
y axis (µm)
C
MDA-MB-231
WT sicon siMCAK
90
42
MCAK
β-actin
1 2 3
D
MDA-MB-231 sicon
MDA-MB-231 WT
MDA-MB-231 siMCAK
E
MDA-MB-231
accumulated distance (µM)
velocity (µM/min)
directionality (d/D)
n.s.
F
HeLa
G
H
open area in %
WT
sicon
siMCAK
(h)
I
J
HeLa
0 h
12 h
18 h
5 h
10 h
15 h

Figure S3: downregulation or overexpression of MCAK leads to reduced motility and migration

(A) The gene level of *KIF2C* (MCAK) was analyzed in RPE cells treated with siRNA against MCAK (siMCAK). Glyceraldehyde-3-phosphate dehydrogenase (GAPDH) served as endogenous control. The data are based on three independent experiments and presented as RQ with minimum and maximum range and statistically analyzed. RQ: relative quantification of the gene expression. **(B)** Representative trajectories of RPE cells with non-treatment, sicon or siMCAK (individual cells n = 30) are shown. **(C and J)** Western Blot analysis of MCAK protein level in MDA-MB-231 (C) and HeLa (J) cells treated with siMCAK. GAPDH served as loading control. **(D)** Representative trajectories of non-treated pr treated MDA-MB-231 cells (individual cells n = 30) are shown. (**E and F**) Random motility of siRNA treated cells was analyzed. MDA-MB-231 (E) and HeLa (F) cells were evaluated for accumulated distance (1st), velocity (2nd), and directionality (3rd). The results are from three independent experiments, and shown as scatter plots with variations. (**G-I**) Wound healing/migration assays were performed with HeLa (G) and MDA-MB-231 cells (H and I), and images were taken at indicated time points to document the migration front. (G and I). White dashed lines depict the migration fronts. Scale: 300 µm. Quantification of the open area between both migration fronts at various time points (H). The cell-free area at 0 h was assigned as 100%. The results are from three independent experiments and presented as mean ± SEM. Unpaired Mann-Whitney *U*-test was used in E and F. $**p < 0.01$, $***p < 0.001$. Student's t-test was used in H. $*p < 0.05$.

Fig. S4

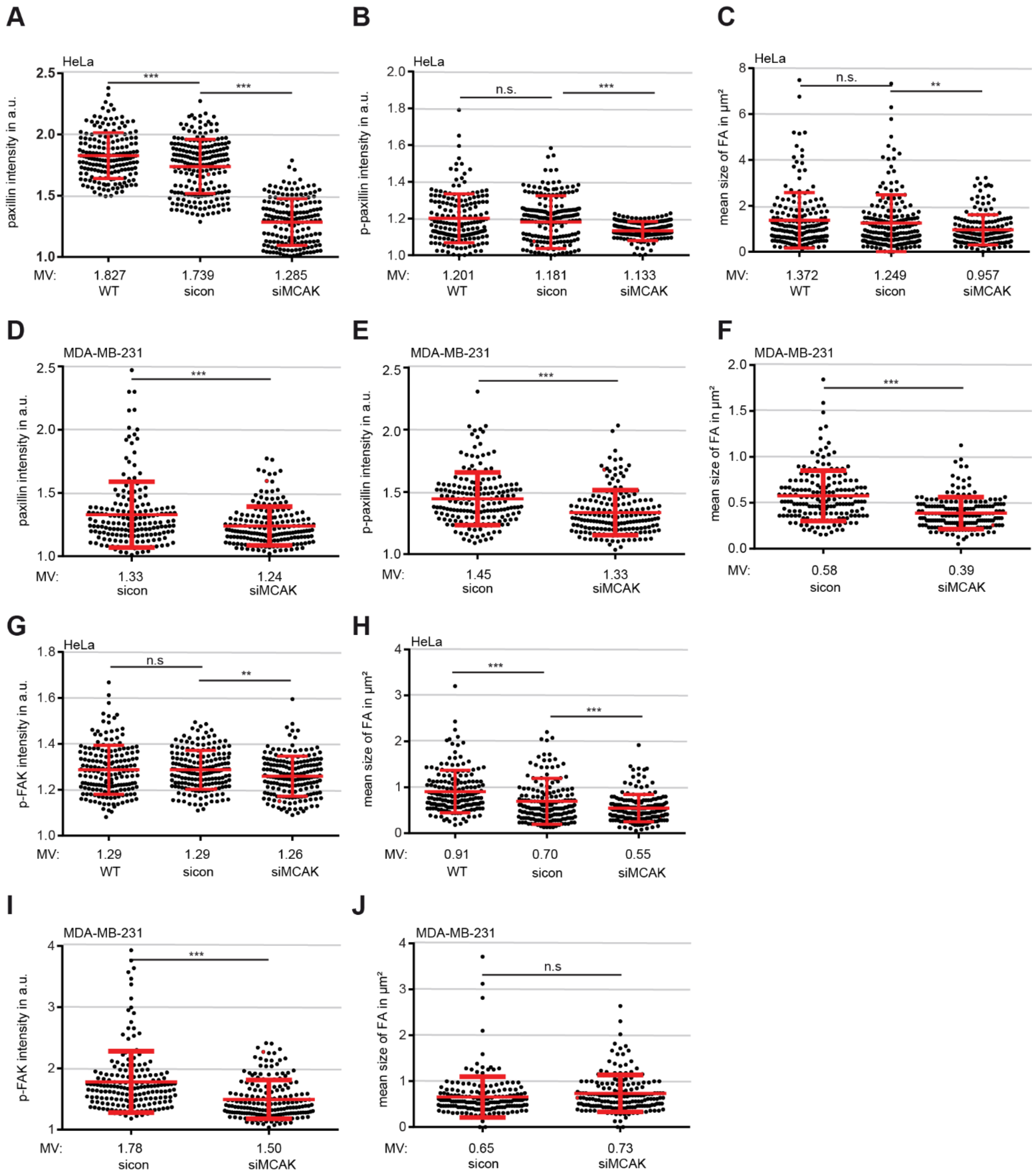
A
HeLa
paxillin intensity in a.u.
***
***
MV: 1.827 WT
1.739 sicon
1.285 siMCAK
B
HeLa
p-paxillin intensity in a.u.
n.s.
***
MV: 1.201 WT
1.181 sicon
1.133 siMCAK
C
HeLa
mean size of FA in µm²
n.s.
**
MV: 1.372 WT
1.249 sicon
0.957 siMCAK
D
MDA-MB-231
paxillin intensity in a.u.
***
MV: 1.33 sicon
1.24 siMCAK
E
MDA-MB-231
p-paxillin intensity in a.u.
***
MV: 1.45 sicon
1.33 siMCAK
F
MDA-MB-231
mean size of FA in µm²
***
MV: 0.58 sicon
0.39 siMCAK
G
HeLa
p-FAK intensity in a.u.
n.s
**
MV: 1.29 WT
1.29 sicon
1.26 siMCAK
H
HeLa
mean size of FA in µm²
***
***
MV: 0.91 WT
0.70 sicon
0.55 siMCAK
I
MDA-MB-231
p-FAK intensity in a.u.
***
MV: 1.78 sicon
1.50 siMCAK
J
MDA-MB-231
mean size of FA in µm²
n.s
MV: 0.65 sicon
0.73 siMCAK

Figure S4: Suppression of MCAK by siRNA deregulates FA proteins and their phosphorylation status

(**A and F**) HeLa and MDA-MB-231 cells were transfected with siRNA against MCAK (siMCAK) and stained for paxillin (green) and p-paxillin (red), or FAK (green) and p-FAK (red) for fluorescence microscopy. (**A-F**) The mean gray intensity of the outlined paxillin (A and D), p-paxillin (B and E) signals and the signal size of paxillin (C and F) were quantified in HeLa (A-C) and MDA-MB-231 (E-F) cells (100 FAs measured in each experiment). The results are based on three independent experiments and presented as scatter plot showing mean ± SEM. (**G-J**) The mean fluorescence intensity of FAK (G and I) and p-FAK (H and J) signals were quantified in HeLa (G and H) and MDA-MB-231 (I and J) cells (100 FAs measured in each experiment). The results are based on three independent experiments and presented as scatter plot showing mean ± SEM. Unpaired Mann-Whitney *U*-test was used. $*p < 0.05$, $**p < 0.01$ and $***p < 0.001$.

Fig. S5

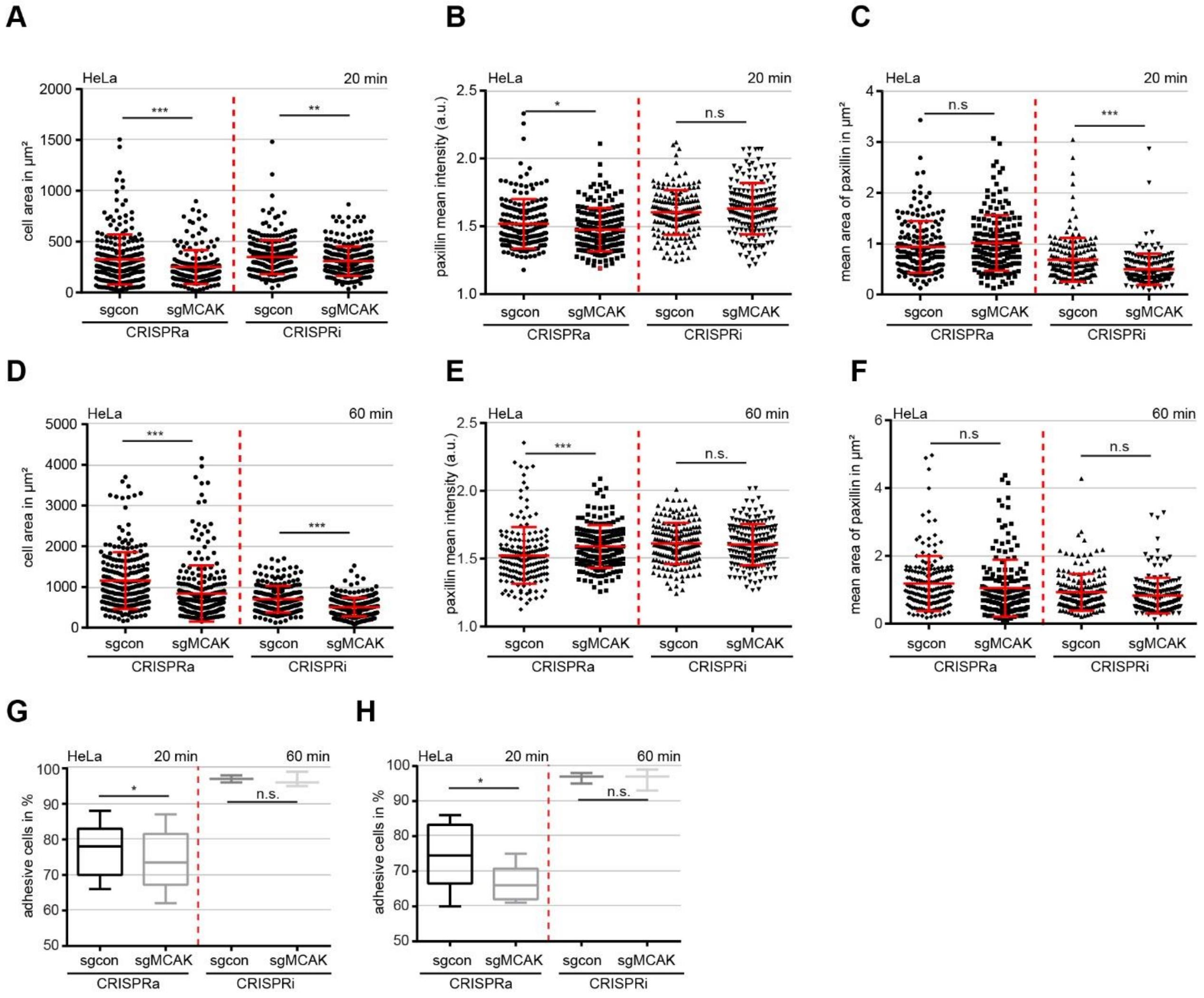

Figure S5: Downregulation or upregulation of MCAK in HeLa CRISPRa/I cells impairs the cell spreading and adhesion capacity.

(**A and D**) Quantification of cell size of HeLa CRISPRi/a sgcon and sgMCAK cells 20 min (A) and 60 min (D) after reseeding. The results are based on three independent experiments and presented as scatter plots showing mean ± SEM (180 cells). (**B and E**) Quantification of the mean fluorescence intensity of paxillin after 20 min (B) and 60 min (E) after reseeding (60 FAs measured in each experiment). The results are based on three independent experiments and presented as scatter plot showing mean ± SEM. (**C and F**) Measurement of the paxillin signal size after 20 min (C) and 60 min (F) reseeding (60 Fas measured in each

experiment). The results are based on three independent experiments and presented as scatter plots showing mean ± SEM. (**G and H**) Quantified percentage of fully re-attached cells after 20 min (G) and 60 min (H) (100 cells). The results are based on three independent experiments and presented as scatter plots showing mean ± SEM. Unpaired Mann-Whitney *U*-test for (D-I). Student's t-test for (J and K). $*p < 0.05$, $**p < 0.01$ and $***p < 0.001$.

Fig. S6

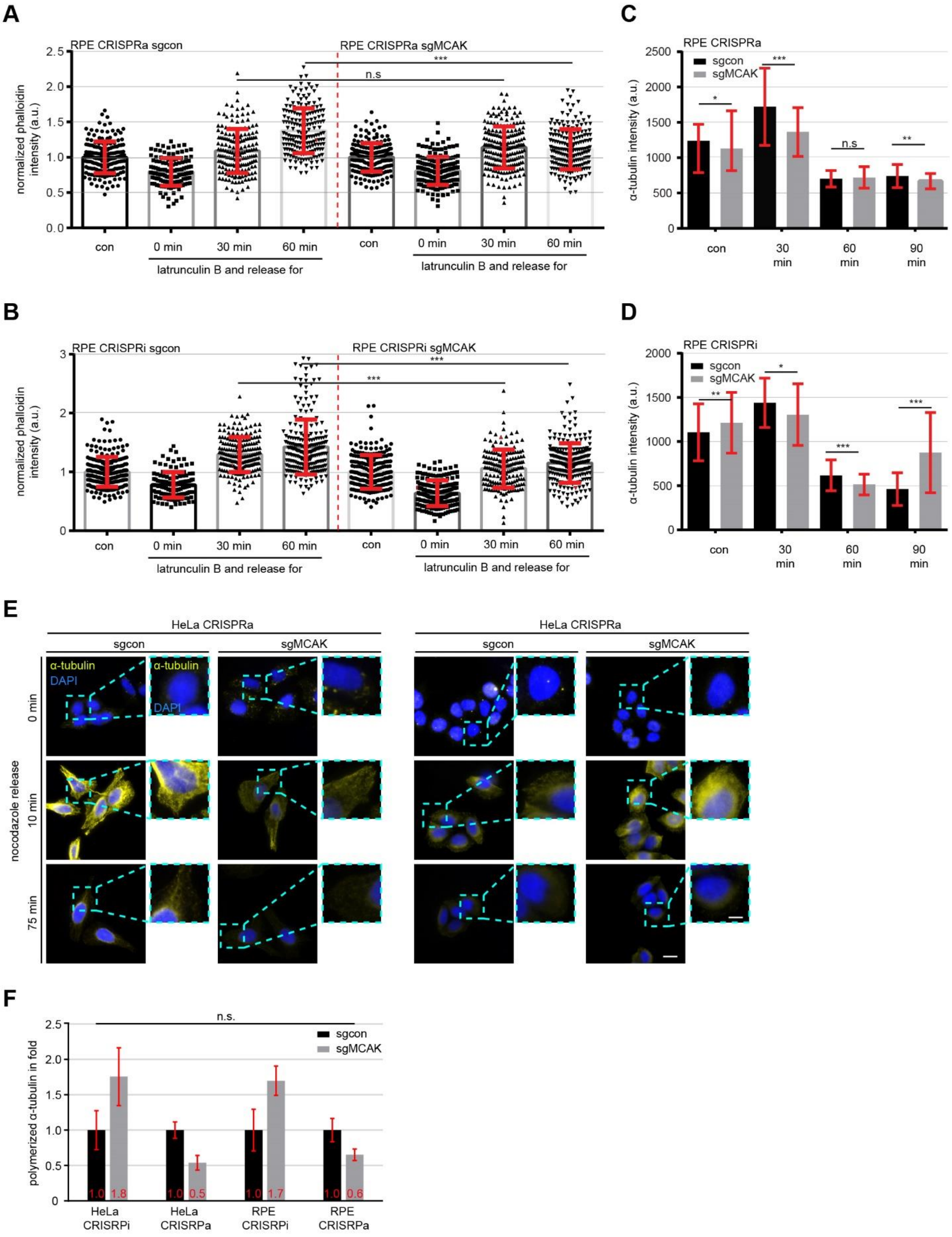
A
RPE CRISPRa sgcon
RPE CRISPRa sgMCAK
normalized phalloidin intensity (a.u.)
n.s
***
con
0 min
30 min
60 min
latrunculin B and release for
B
RPE CRISPRi sgcon
RPE CRISPRi sgMCAK
C
RPE CRISPRa
sgcon
sgMCAK
α-tubulin intensity (a.u.)
D
RPE CRISPRi
90 min
E
HeLa CRISPRa
α-tubulin
DAPI
nocodazole release
10 min
75 min
F
polymerized α-tubulin in fold
n.s.
HeLa CRISRPi
HeLa CRISRPa
RPE CRISRPi
RPE CRISRPa

Figure S6: Deficient actin fiber and microtubule repolymerization in RPE CRISPRa/i cells treated with latrunculin B.

(**A-D**) RPE CRISPRa/i cells were treated with 200 nM latrunculin B for 90 min and released into fresh medium for indicated time periods. The cells were stained for F-actin (phalloidin, red) (A and B) and α-tubulin (green) (C and D). (**A and C**) The mean fluorescence intensity of F-actin (phalloidin) per cell (30 cells per condition) was quantified in RPE CRISPRa (A) and RPE CRISPRi (B) cells. The results are based on three independent experiments and presented as scatter plots showing mean ± SEM, a.u., arbitrary units. (**C and D**) The microtubule repolymerization dynamics was quantified by measuring the mean fluorescence intensity of α-tubulin (30 cells per condition) in RPE CRISPRa (C) and RPE CRISPRi (D) cells. The results are based on three independent experiments and presented as bar graphs showing mean ± SEM. Unpaired Mann-Whitney *U*-test for (A and B). Student's t-test for (C and D). $*p < 0.05$ and $***p < 0.001$. (**E**) HeLa CRISPRi/a cells were treated with 10 μM nocodazole for 5 h and released into fresh medium for indicated time points. HeLa CRISPRa (1st panel) and CRISPRi (2nd panel) cells were stained for α-tubulin (yellow) and DNA (DAPI, blue). Representatives of MT disassembly and assembly during 0 to 75 min are shown. Scale: 25 μm, inset scale: 12.5 μm. (**F**) In HeLa CRISPR a/i cells (1st graph) and RPE CRISPRa/i cells (2nd graph) the polymerized α-tubulin content was measured *in vivo.;* Cellular soluble tubulin was pre-extracted in a MT-stabilizing buffer. Resuspended cells were fixed and stained for α-tubulin with a specific mouse monoclonal antibody and FITC-conjugated rabbit anti-mouse antibody. The α-tubulin content of these cells was analyzed using a FACSCalibur™. The content in CRISPRa/i sgcon cells was assigned as 100%. The results are based on three independent experiments and presented as mean ± SEM. Student's t-test was used. $*p < 0.05$ and $***p < 0.001$.

Additional file 1: original WBs Figure 1

Fig. 1C **PLK1**

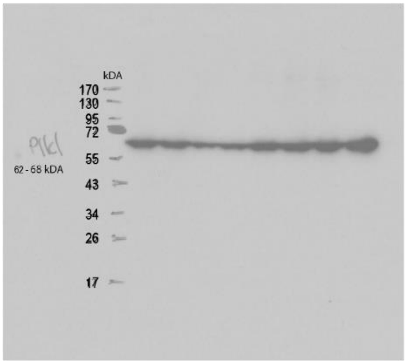

Fig. 1C **Aurora A**

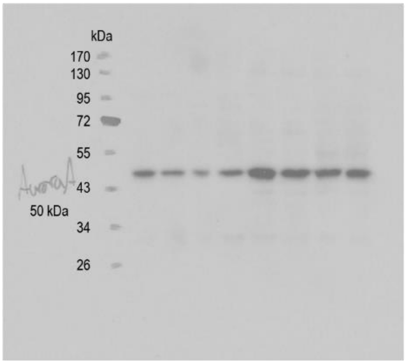

RPE	CRISPRa		CRISPRi	
28.10.2021	sgcon	sgMCAK	sgcon	sgMCAK
GAPDH	143.33	140.19	131.33	151.82
GAPDH - BG	50.11	46.97	38.11	58.6
Cyclin B1	127.56	128.41	126.5	133.97
Cyclin B1- BG	34.34	35.19	33.28	40.75
RATIO	0.23958697	0.25101648	0.25340745	0.26840996
Normalized to control	1	1.04770506	1	1.05920312

HeLa	CRISPRa		CRISPRi	
28.10.2021	sgcon	sgMCAK	sgcon	sgMCAK
GAPDH	129.02	115.42	139.01	133.46
GAPDH - BG	35.8	22.2	45.79	40.24
Cyclin B1	150.35	154.77	148.14	141.89
Cyclin B1- BG	57.13	61.55	54.92	48.67
RATIO	0.44279957	0.5332698	0.39507949	0.36467856
Normalized to control	1	1.20431418	1	0.92305109

Fig. 1C **GAPDH**

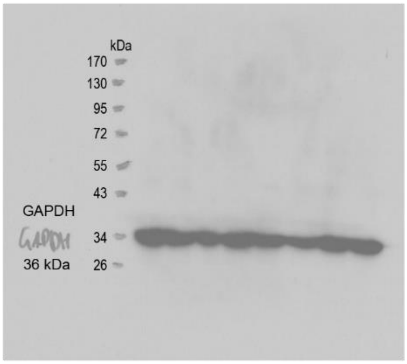

Fig. 1C **Cyclin B1**

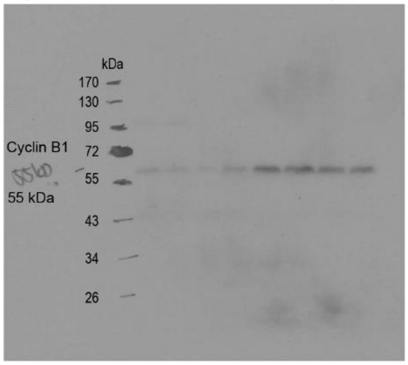

RPE	CRISPRa		CRISPRi	
28.10.2021	sgcon	sgMCAK	sgcon	sgMCAK
GAPDH	143.33	140.19	131.33	151.82
GAPDH - BG	50.11	46.97	38.11	58.6
Plk1	189	172.43	160.42	155.43
Plk1 - BG	95.78	79.21	67.2	62.21
RATIO	0.6682481	0.5650189	0.51168811	0.40976156
Normalized to control	1	0.84552265	1	0.80080336

Fig. 1C HeLa **MCAK/KIF2C**

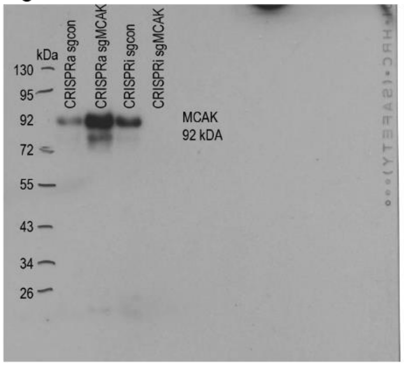

Fig. 1C **MCAK/KIF2C & β-actin**

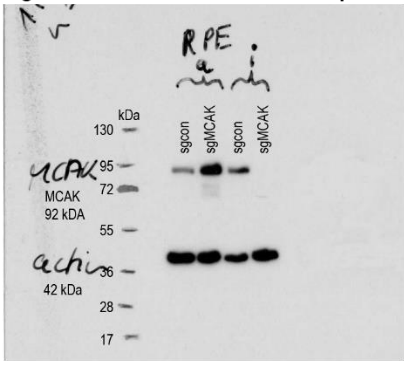

HeLa	CRISPRa		CRISPRi	
28.10.2021	sgcon	sgMCAK	sgcon	sgMCAK
GAPDH	129.02	115.42	139.01	133.46
GAPDH - BG	35.8	22.2	45.79	40.24
Plk1	148.74	160.87	174.87	180.18
Plk1 - BG	55.52	67.65	81.65	86.96
RATIO	0.43032088	0.58612026	0.58736782	0.651581
Normalized to control	1	1.36205395	1	1.10932363

Fig. 1C **β-actin**

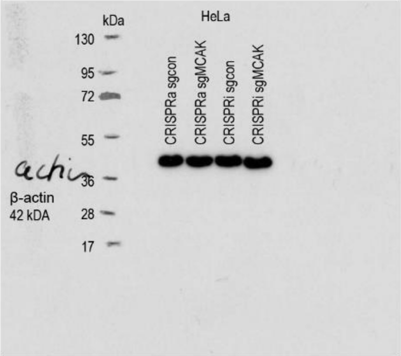

RPE	CRISPRa		CRISPRi	
28.10.2021	sgcon	sgMCAK	sgcon	sgMCAK
Actin	87.79	86.12	65.72	81.67
Actin - BG	66.1	64.43	44.03	59.98
MCAK	36.42	71.73	44.1	22.55
MCAK - BG	14.73	50.04	22.41	0.86
RATIO	0.16778676	0.5810497	0.34099209	0.01053018
Normalized to control	1	3.46302464	1	0.03088102

RPE	CRISPRa		CRISPRi	
28.10.2021	sgcon	sgMCAK	sgcon	sgMCAK
GAPDH	143.33	140.19	131.33	151.82
GAPDH - BG	50.11	46.97	38.11	58.6
Aurora A	129.24	124.69	110.6	118.49
Aurora A - BG	36.02	31.47	17.38	25.27
RATIO	0.25130817	0.22448106	0.13233838	0.16644711
Normalized to control	1	0.89325015	1	1.25773871

HeLa	CRISPRa		CRISPRi	
28.10.2021	sgcon	sgMCAK	sgcon	sgMCAK
Actin	133.08	139.35	130.23	125.86
Actin - BG	36.87	43.14	34.02	29.65
MCAK	60.54	114.75	82.66	46.51
MCAK - BG	18.74	72.95	40.86	4.71
RATIO	0.14081755	0.52350197	0.31375259	0.03742253
Normalized to control	1	3.71759032	1	0.11927402

HeLa	CRISPRa		CRISPRi	
28.10.2021	sgcon	sgMCAK	sgcon	sgMCAK
GAPDH	129.02	115.42	139.01	133.46
GAPDH - BG	35.8	22.2	45.79	40.24
Aurora A	170.61	159.12	150.05	148.16
Aurora A - BG	77.39	65.9	56.83	54.94
RATIO	0.59982948	0.57095824	0.40881951	0.41165892
Normalized to control	1	0.95186758	1	1.0069454
